# DOTA‐grafted Cationic Polymers Behaving as Powerful Macromolecular Resistance‐reversal Agents (MRRAs) Combating Against New Delhi Metallo‐β‐lactamase (NDM)‐producing Bacteria

**DOI:** 10.1002/advs.202519056

**Published:** 2026-03-05

**Authors:** Ruixue Wang, Jian Zhang, Yun li, Liping Qiao, Zhuorui Dong, Dandan Cui, Peirong Bai, Liping Li, Bing Cao, Ruiping Zhang

**Affiliations:** ^1^ Third Hospital of Shanxi Medical University Shanxi Bethune Hospital Shanxi Academy of Medical Sciences Tongji Shanxi Hospital Taiyuan China; ^2^ The Radiology Department of Shanxi Provincial People's Hospital Affiliated to Shanxi Medical University Taiyuan China; ^3^ Key Laboratory of Interface Science and Engineering in Advanced Materials Ministry of Education Taiyuan University of Technology Taiyuan China; ^4^ School of Basic Medical Sciences Shanxi Medical University Taiyuan China

**Keywords:** antibacterial, cationic polymer, chelation, drug resistance, NDM‐producing bacteria

## Abstract

New Delhi metallo‐β‐lactamase (NDM)‐producing bacteria have posed a significant threat to human health. To navigate this tricky problem, numerous NDM inhibitors have been explored, showing great antibacterial synergy. But there are still be some problems to be tackled, such as suboptimal efficacy, rapid resistance evolution, and high cytotoxicity. Herein, we engineer two macromolecular resistance‐reversal agents (MRRAs) via pharmacophore integration: linear (MRRAL) and nanostructured (MRRAN) topologies, both coordinating metal ion chelator‐DOTA motif into tunable polycationic scaffolds. And we validate their efficacy through 4 clinically isolated NDM‐producing pathogens. MRRAs present excellent effect boosting meropenem (MEM) to fight against those superbugs with the fractional inhibitory concentration indices much less than 0.5. Notably, the nanostructured MRRAN exhibited superior potency compared to its linear counterpart MRRAL under the same concentration of the active group of “DOTA.” Mechanistic studies revealed that MRRAN could achieve bacterial membrane targeting through charge‐mediated accumulation, exerting triple‐action synergistic mechanisms: 1) physical membrane disruption through powerful local cationic surface interactions and hydrophobic force, 2) precise Zn^2^^+^ depletion from membrane‐associated NDM, and 3) localized DOTA enrichment for enhanced metalloenzyme inhibition. Our findings established a paradigm for rational design of macromolecule metalloenzyme inhibitors through spatial organization of pharmacophores, offering a promising therapeutic strategy against NDM‐mediated carbapenem resistance.

## Graphical abstract

Two macromolecular resistance‐reversal agents (MRRAs)—linear (MRRA_L_) and nanostructured (MRRA_N_) polymers were fabricated by integrating DOTA motifs into tunable polycationic scaffolds. MRRAs exhibit superior antibacterial potency once combined with meropenem (MEM) through (i) bacterial membrane disruption via localized cationic charge and hydrophobicity, (ii) Zn^2+^ depletion to inactivate NDM enzymes, and (iii) sustained metalloenzyme inhibition via DOTA enrichment, offering a promising therapeutic strategy against NDM‐mediated carbapenem resistance.

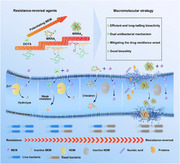

## 1 Introduction

Bacterial resistance is one of the world's top 10 health concerns, severely threatening human life and leading to a heavy economic burden worldwide [1, 2, 3]. The emergence of New Delhi metallo‐β‐lactamase (NDM)‐producing bacteria, which present resistance to nearly all the clinically available antibiotics, including the last‐resort carbapenems, has dramatically exacerbated this issue due to the rapid resistance evolution and global dissemination [4, 5, 6]. However, the new drug development pipeline has been almost depleted in the last few decades, barely met the clinical needs [7, 8]. There is a pressing desire to exploit some innovative strategies to combat those deadly superbugs.

Combination therapy, revitalizing or repurposing the existing drugs, has been perceived as a promising approach to combat drug resistance [9, 10, 11]. Considering that the NDM‐enabled carbapenems degradation is the main reason for therapy failure, the NDM‐targeting inhibitors are supposed to be potential adjuvants restoring the bioactivity of inactivated antibiotics [12, 13]. Because NDM is a metalloenzyme with two Zn^2+^ acting as the cofactor in the active pocket, metal‐chelators have been discovered as the most potent NDM inhibitors to fight against NDM‐producing bacteria by competing Zn^2+^in the active center [12, 14]. Over the past decades, plenty of synthetic compounds and natural products with metal chelating function (e.g. Ethylenediaminetetraacetic Acid (EDTA), tetrakis‐(2‐pyridylmethyl)ethylenediamine (TPEN), di‐(2‐picolyl)amine (DPA), 1,4,7‐triazacyclononane‐1,4,7‐triacetic acid (NOTA), 1,4,7,10‐tetraazacyclododecane‐1,4,7,10‐tetraacetic acid (DOTA), and aspergillomarasmine A (AMA)) have been exploited, attempting to reverse the drug resistance of NDM‐producing bacteria [15, 16, 17], but numerous challenges still be remained. Rapid blood clearance, poor efficacy, limited availability, fast resistance evolution, and multiple‐dose exposure account for the awkward problems to be solved [18, 19, 20, 21]. Particularly, traditional small molecular metal chelators can indiscriminately access the lesion location and normal tissues to deprive Zn^2+^ for many physiological enzymes, leading to serious off‐target toxicity. Therefore, some kind of much more efficient and safer NDM inhibitor is essentially required.

For the last few years, the macromolecular polycations have emerged as transformative materials to combat drug resistance, owing to their unique multifunctional advantages: (1) as membrane‐lytic agents, they not only boost drug permeability by bacterial membranes compromise, but also exhibit a lower propensity for inducing resistance development compared to conventional antibiotics; (2) Their prolonged blood circulation kinetics, particularly in polymeric micelle formulations, enable enhanced lesion targeting and sustained retention to amplify therapeutic efficacy; (3) The intrinsic structural tunability and facile functionalization of these macromolecules permit precise control over physicochemical properties and multi‐mechanism antimicrobial functionalities [22, 23, 24, 25]. Despite these merits, the development for macromolecular polycation‐based NDM inhibitors remains underexplored. To our knowledge, no systematic studies have been reported on rationally engineered polycationic architectures that integrate NDM inhibition with pathogen‐specific targeting and multimodal bactericidal actions.

**SCHEME 1:**
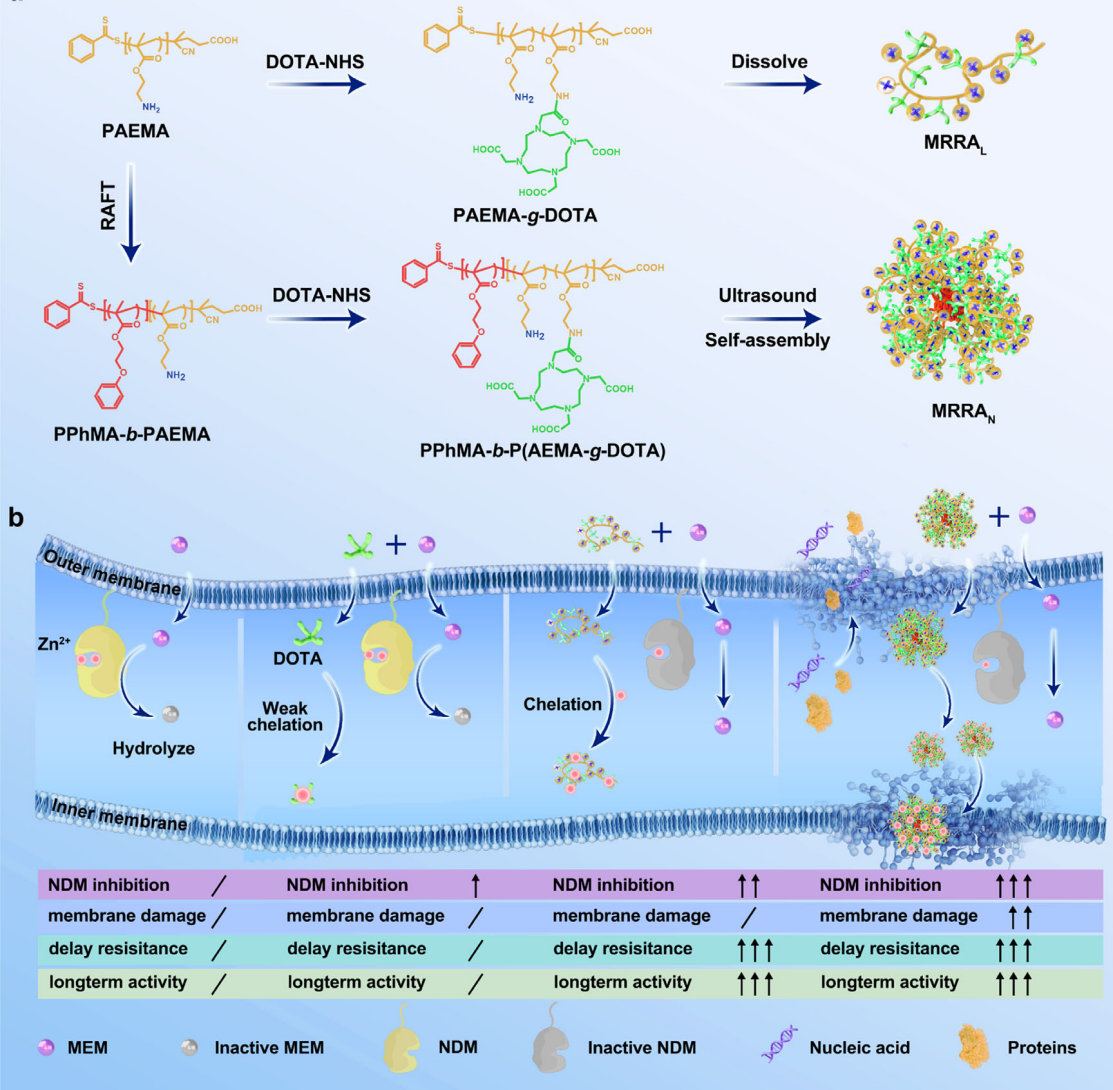
(a) Schematic illustration for the synthesis of polycation‐based macromolecular resistance‐reversal agents (MRRAs): linear (MRRA_L_) and nanostructured (MRRA_N_) topologies. (b) The associated mechanisms of MRRAs to boost MEM fighting against NDM‐producing bacteria. MRRAs have an enhanced capacity of chelation Zn^2+^, which is attributed to the multivalent effect of active groups of “DOTA.” In addition, MRRA_N_ could physical damage the cell membrane mediated by electrostatic complementation and hydrophobic forces.

In this study, we engineer two macromolecular resistance‐reversal agents (MRRAs) through strategic integration of metal‐chelating pharmacophores into architecturally distinct polycationic platforms: linear MRRA_L_ and nanoparticle‐structured MRRA_N_. The design capitalizes on dual mechanisms: DOTA motifs function as dynamic zinc chelators to disrupt NDM metalloenzyme activity through Zn^2^^+^ sequestration, while the cationic scaffolds (particularly the nanoscale MRRA_N_) mechanically compromise bacterial membrane integrity via electrostatic interactions with anionic microbial surfaces. This bifunctional action synergistically enhances membrane permeability while suppressing NDM‐mediated resistance (Scheme 1). Systematic evaluation through 4 clinically significant pathogens: NDM‐producing *Enterobacter cloacae* (*E. cloacae*), NDM‐producing *Escherichia coli* (*E. coli*), NDM‐producing *Klebsiella pneumoniae* (*K. pneumoniae*), and pan‐drug‐resistant *Acinetobacter baumannii* (*A. baumannii*), demonstrating that both MRRA_L_ and MRRA_N_ self‐assembled by DOTA‐grafted amphiphilic polymer behaved remarkable resistance reversal capabilities for restoring MEM efficiency by 48–96 fold at 5.60 µm of polymers, outperforming DOTA small molecule, achieving equivalent NDM inhibition at 9‐fold lower doses with preventing resistance development‐advantages as well as maintaining low cytotoxicity and long‐term efficacy. Notably, the nanostructured MRRA_N_ is superior to its linear counterpart MRRA_L_ in the case of normalized concentration of active group “DOTA,” which was attributed to its enhanced local cationic density and optimized DOTA spatial distribution. Additionally, the polycationic nanoparticle architecture with nano‐scale size shares more therapeutic advantages: 1) Size‐dependent enrichment at infection sites via enhanced permeation and retention (EPR) effects [26, 27, 28], 2) Selective bacterial membrane targeting and disrupting through charge complementarity and hydrophobic interactions (avoiding neutral mammalian cells) [29, 30, 31], and 3) Improved pharmacophore local payload delivery and expose capability. These features position MRRA_N_ as a more promising candidate for targeted anti‐infective therapy with minimized off‐target effects and powerful efficacy.

## 2 Results and Discussion

### 2.1 Synthesis and Characterization of MRRA_L_ and MRRA_N_

We first polymerized 2‐aminoethyl methacrylate hydrochloride (AEMA) to obtain linear polycationic scaffold PAEMA by classical reversible addition‐fragmentation chain transfer polymerization (RAFT) method [32]. The average polymerization degree of AEMA was determined to be 25 by Proton Nuclear Magnetic Resonance Spectroscopy (^1^H NMR) analysis, and the polymer was labeled as PAEMA_25_ (Figure S1). PAEMA_25_ presents a positive charge due to the 25 amino groups (‐NH_2_) inside the molecular structure acting as the final linear polycationic scaffold, shorted as PS_L_. Next, DOTA‐NHS ester was reacted with PAEMA_25_ by amide reaction to afford DOTA‐grafted PAEMA. The average degree of DOTA grafted to PAEMA_25_ was determined to be 9, and the product was labeled as PAEMA_16_‐*g*‐DOTA_9_ (Figure S2), which acted as the linear macromolecular resistance‐reversal agent (MRRA_L_). To fabricate its nanostructured analogues with a three‐dimensional structure, the amphiphilic polymer PPhMA‐*b*‐PAEMA_25_ was then obtained by block polymerizing 2‐phenoxyethyl methacrylate (PhMA) using PAEMA_25_ as macro‐chain transfer agent. The polymerization degree of PhMA was confirmed to be 33. And the final amphiphilic polymer was labeled as PPhMA_33_‐*b*‐PAEMA_25_ (Figure S3), which could be self‐assembled under ultrasonic to form a polycationic nanoparticle scaffold (PS_N_). Then, DOTA‐NHS ester was grafted to PPhMA_33_‐*b*‐PAEMA_25_ to afford PPhMA_33_‐*b*‐P(AEMA_17_‐*g*‐DOTA_8_), coupled with 8 molecules of DOTA to each amphiphilic polymer PPhMA_33_‐*b*‐PAEMA_25_ (Figure S4). The above four polymers were also tested by Gel Permeation Chromatography (GPC), all of them have a low polydispersity index (PDI) (less than 1.4), which implied the uniform molecular weight distribution (Figures S5–S8). The structure parameters of all above four polymers, including degree of polymerization, molecular weight, and molecular weight distributions, were listed in Table S1. Then, PPhMA_33_‐*b*‐P(AEMA_17_‐*g*‐DOTA_8_) was self‐assembled under ultrasonic to form nanoparticles with three‐dimensional structures and named as nanostructured macromolecular resistance‐reversal agent (MRRA_N_), which has a hydrophobic core and a hydrophilic polycationic shell grafted with DOTA molecules.

**FIGURE 1:**
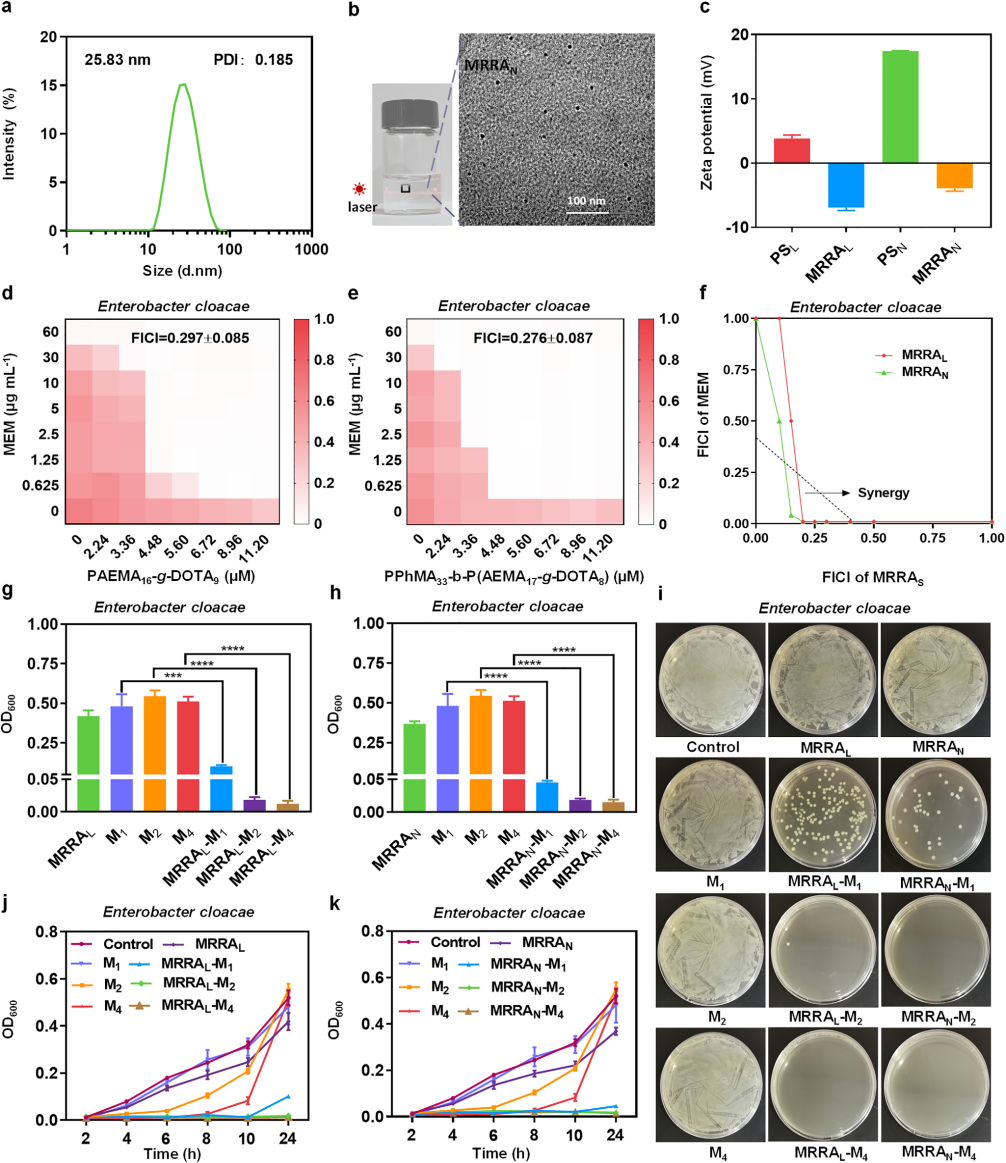
(a) Hydrodynamic diameter distributions of MRRA_N_. (b) Tyndall phenomenon and TEM image of MRRA_N_. Scale bar = 100 nm. (c) Zeta potentials of PS_L_, MRRA_L_, PS_N,_ and MRRA_N_. (d, e) The checkerboard assays to observe the synergetic effect between MEM and MRRA_L_ (PAEMA_16_‐*g*‐DOTA_9_) / MRRA_N_ (self‐assembled by PPhMA_33_‐*b*‐P(AEMA_17_‐*g*‐DOTA_8_)) against NDM‐producing *E. cloacae*. (f) Isobolograms of the co‐therapy of MEM with MRRAs against NDM‐producing *E. cloacae*. The triangle area represents the synergistic area. (g, h) The OD_600_ in groups treated with or without MRRA_L_ (5.60 µm) / MRRA_N_ (self‐assembled by 5.60 µm of PPhMA_33_‐*b*‐P(AEMA_17_‐*g*‐DOTA_8_)) combined with different concentrations of MEM (1 µg mL^−1^ (M_1_), 2 µg mL^−1^ (M_2_), and 4 µg mL^−1^ (M_4_)). (i) The corresponding plate counting results under the same treatment conditions. (j, k) The corresponding time‐dependent growth curves of NDM‐producing *E. cloacae* under the same treatment conditions. Data are presented as mean ± SD (*n* = 3); ^***^, and ^****^ indicate *p* < 0.001, and *p* < 0.0001 respectively.

Dynamic light scattering (DLS) results showed that the average hydrodynamic diameter (Z‐average) of MRRA_N_ was about 25 nm (Figure 1a) based on the intensity distribution, and exhibited low polydispersity (PDI = 0.185), demonstrating a relatively uniform size distribution. The main autocorrelation plots were presented in Figure S9. After laser irradiation, the MRRA_N_ solution exhibited light path (Tyndall phenomenon), demonstrating that the colloidal solution was successfully fabricated. Furthermore, the transmission electron microscopy (TEM) confirmed that MRRA_N_ had a regular small spherical shape with an average size around 10 nm, which was consistent with DLS results (Figure 1b), owing to the hydrodynamic diameter is typically larger than the actual size. In addition, the Zeta potentials of PS_L_ and PS_N_ were decreased after grafting DOTA from the original +3.81 ± 0.56 mV and +17.40 ± 0.11 mV to ‐6.93 ± 0.44 mV and ‐3.90 ± 0.47 mV, respectively (Figure 1c). All of the dates demonstrated the successful grafting of DOTA.

### 2.2 MRRA_L_ and MRRA_N_ Revitalize the Bioactivity of MEM Fighting against NDM‐Producing *E. cloacae*

MEM, one of the most widely used carbapenem antibiotics, which are the last resort for gram‐negative bacteria, while having weak or no efficacy for NDM‐producing bacteria had been taken as a model drug to be evaluated. The NDM‐producing *E. cloacae*, the most popular NDM‐producing bacteria in the local region, was taken as the case strain at first. The strain was screened by the tests of modified carbapenem inactivation method (mCIM) and EDTA‐carbapenem inactivation method (eCIM) according to the CLSI M100‐32 (Figure S10). Then, we confirmed the NDM gene based on the fluorescent PCR method conducted on GeneXpert Infinity Systems (Figure S11). Standard checkboard assay was performed to assess the synergy between MEM and MRRA_L_/MRRA_N_. Throughout the experiment, the bacteria receiving MRRA_L_‐MEM and MRRA_N_‐MEM co‐therapy exhibited much more powerful suppression than each monotherapy of MEM, MRRA_L_/MRRA_N_. When MRRA_L_ (5.60 µm) or MRRA_N_ (self‐assembled by 5.60 µm of PPhMA_33_‐*b*‐P(AEMA_17_‐*g*‐DOTA_8_) was combined, the MIC_MEM_ reduced substantially from the original 60 µg mL^−1^ to 1.25 µg mL^−1^ or 0.625 µg mL^−1^. Meanwhile, the fractional inhibitory concentration index (FICI) was evaluated. FICI < 0.5 indicates synergy, whereas FICI = 0.5∼4 additive, and FICI > 4 antagonism [33]. A typical synergistic effect was observed between MEM and MRRA_L_/MRRA_N_ with FICI = 0.297 ± 0.085 and 0.276 ± 0.087, respectively (Figure 1d, e). As illustrated in isobolograms (Figure 1f), the data points in the triangle area (synergy line and X, Y axis) demonstrated the synergy between the two components. Compared with two previously reported macromolecular NDM inhibitors, PAMAM [34], a hyperbranched polycationic with FICI of around 0.312. Thanatin [35], an inducible 21‐residue insect peptide with FICI ranging from 0.508 to 0.625. MRRA_L_/MRRA_N_ has a comparable or superior synergistic effect with MEM fighting against NDM‐producing bacteria. Whereas, MRRA_L_/MRRA_N_ shares much more higher biosafety and facile synthesis through pharmacophore immigration strategy, facilitating large‐scale production at low cost [36, 37]. In conclusion, MRRA_L_ and MRRA_N_ are supposed to be two promising macromolecular‐based NDM inhibitors to repurpose MEM as a potent antibiotic with good clinical translational prospects.

Then, MRRA_L_ (5.60 µm) / MRRA_N_ (self‐assembled by 5.60 µm of PPhMA_33_‐*b*‐P(AEMA_17_‐*g*‐DOTA_8_) was selected to be combined with various doses of MEM (1, 2^,^ and 4 µg mL^−1^ shorted by M_1_, M_2_, and M_4_), combating against NDM‐producing *E. cloacae* (MIC_MEM_ = 60 µg mL^−1^) for better understanding the capacity of MRRA_L_/MRRA_N_ to intensify the bioactivity of MEM. After 24 h incubation, the OD_600_ was significantly lower in MRRA_L_‐MEM and MRRA_N_‐MEM co‐therapy groups than in the monotherapy group of MEM or MRRA_L_/MRRA_N_ alone. And when MEM was taken > 1 µg mL^−1^, the OD_600_ basically remained stable and barely decreased anymore, implying that 2 µg mL^−1^ of MEM was enough to wipe out nearly all the bacteria reaching the minimum bactericidal concentration (MBC).(Figure 1g,h). And this speculation was confirmed by the colony counting method. There was almost no growth on the plates in the co‐therapy groups with 2 or 4 µg mL^−1^ of MEM combined (Figure 1i), suggesting that MRRA_L_ (5.60 µm) or MRRA_N_ (self‐assembled by 5.60 µm of PPhMA_33_‐*b*‐P(AEMA_17_‐*g*‐DOTA_8_)) could remarkably enhance the MEM inhibiting or killing almost all the NDM‐producing *E. cloacae* at very low MEM doses. Lastly, the bacterial growth kinetic curves were recorded to depict the bacterial growth status for both co‐therapy and monotherapy groups. At the beginning of growth, 2 or 4 µg mL^−1^ of MEM exhibited a transient inhibitory effect, respectively, then they failed after 6 or 8 h incubation, and the growth curves began to rise, reaching the same growth status as the control group after 24 h. In contrast, the co‐therapy groups were constantly maintained bacterial suppression throughout the whole observation (Figure 1j,k). Taken together, MRRA_L_/MRRA_N_ was potent in restoring the bioactivity of MEM against NDM‐producing *E. cloacae* with a low dose of polymers (5.60 µm), demonstrating a high probability to reuse MEM to eradicate NDM‐producing bacteria.

### 2.3 Validation of Broad‐Spectrum Activity of the MRRA_L_/MRRA_N_ Enhancing MEM Fighting Against Other Clinically Important Pathogens

**FIGURE 2:**
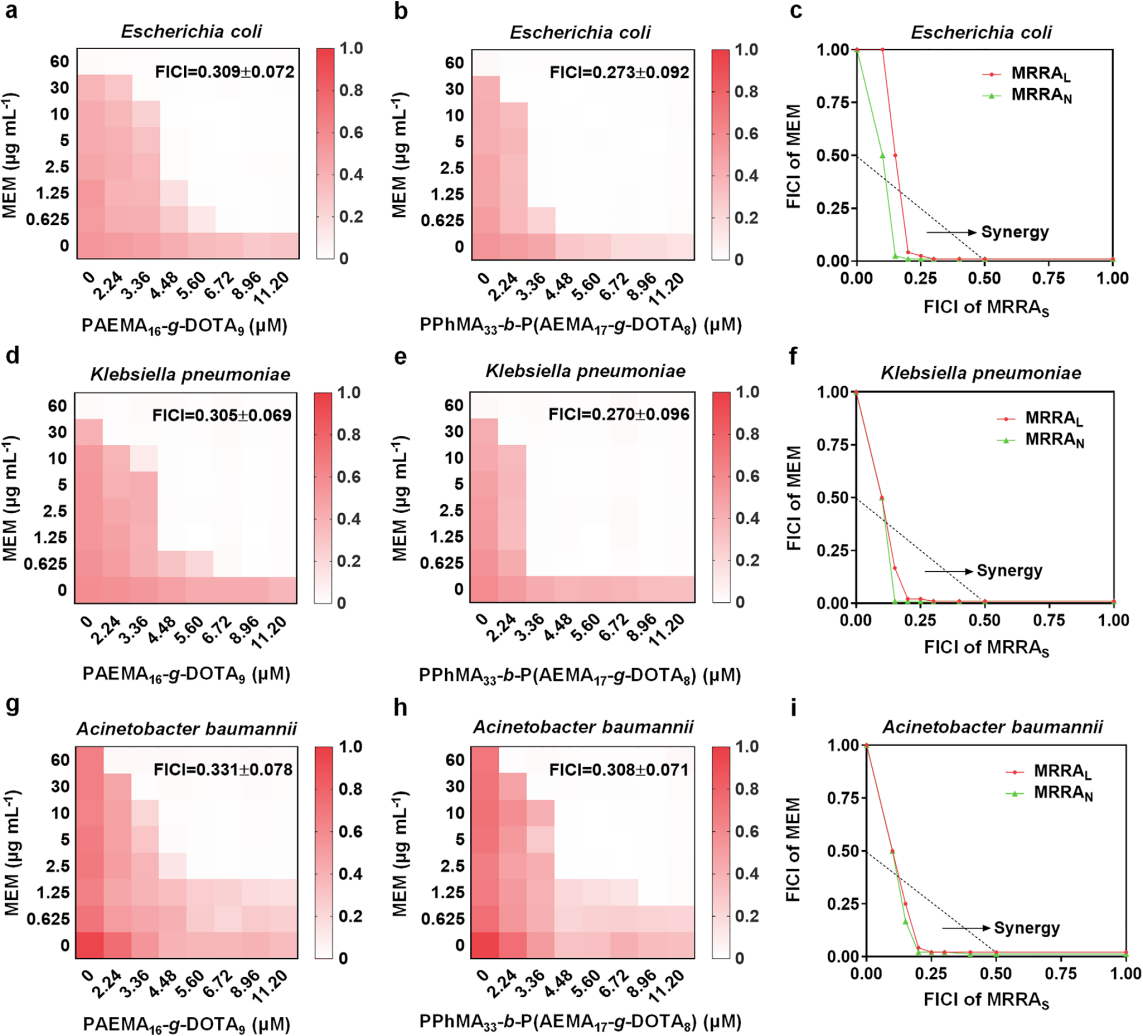
The synergistic effects between MEM and MRRA_L_ (PAEMA_16_‐*g*‐DOTA_9_) / MRRA_N_ (self‐assembled by PPhMA_33_‐*b*‐P(AEMA_17_‐*g*‐DOTA_8_) against NDM‐producing *E. coli*, NDM‐producing *K. pneumoniae*, and pan‐drug‐resistant *A. baumannii*. (a–c) The checkerboard assays and isobolograms to observe the synergetic effect between MEM and MRRA_L_/MRRA_N_ against NDM‐producing *E.coli*. (d–f) The checkerboard assays and isobolograms to observe the synergetic effect between MEM and MRRA_L_/MRRA_N_ against NDM‐producing *K. pneumoniae*. (g–i) The checkerboard assays and isobolograms to observe the synergetic effect between MEM and MRRA_L_/MRRA_N_ against pan‐drug‐resistant *A. baumannii*. The triangle areas in isobolograms for different species represents the synergistic area.

Subsequently, we broadened the antibacterial spectrum to validate the generalizability of MRRA_L_/MRRA_N_ to enhance MEM fighting against other NDM‐producing bacteria. NDM‐producing *E. coli* and NDM‐producing *K. pneumoniae*, two major clinically significant pathogens, were screened and confirmed by the same way as the NDM‐producing *E. cloacae* (Figures S10, S12, S13). As expected, the synergism between MEM and MRRA_L_/MRRA_N_ was also observed in both of the above two strains, with FICI ranging from 0.270 ± 0.096 to 0.309 ± 0.072. While the concentration of PAEMA_16_‐*g*‐DOTA_9_ or PPhMA_33_‐*b*‐P(AEMA_17_‐*g*‐DOTA_8_) reached 5.60 µm, MRRA_L_/MRRA_N_ could reduce the MIC_MEM_ from 60 µg mL^−1^ to 0.625–1.25 µg mL^−1^, potentiating 48–96 times MEM efficacy (Figure 2a–f). In addition, a pan‐drug‐resistant *A. baumannii* isolated from a clinical setting behaving much more severely MEM‐resistant with the MIC = 120 µg mL^−1^ was also tested. Intriguingly, MRRA_L_/MRRA_N_ also could significantly potentiate MEM against pan‐drug‐resistant *A. baumannii* with the FICI = 0.331 ± 0.078 and 0.308 ± 0.071, respectively. After being combined with MRRA_L_ (5.60 µm) / MRRA_N_ (self‐assembled by 5.60 µm of PPhMA_33_‐*b*‐P(AEMA_17_‐*g*‐DOTA_8_), the corresponding MIC_MEM_ could be decreased from the original 120 to 2.5 µg mL^−1^ (Figure 2g–i). When MRRA_L_/MRRA_N_ was combined with different concentrations of MEM (1, 2, and 4 µg mL^−1^, shorted as M_1_, M_2_, M_4_) fighting against all the above strains, the efficacy of co‐therapy was much more superior than monotherapy (Figures S14 and S15). Moreover, the time‐dependent growth curves indicated that it needed at least 4 µg mL^−1^ of MEM to be combined with MRRA_L_ (5.60 µm) / MRRA_N_ (self‐assembled by 5.60 µm of PPhMA_33_‐*b*‐P(AEMA_17_‐*g*‐DOTA_8_) to inhibit the growth of pan‐drug‐resistant *A. baumannii* completely. Whereas, 1–2 µg mL^−1^ of MEM was sufficient to achieve the same effect for NDM‐producing *E. coli* and *K. pneumoniae*, indicating that MRRA_L_/MRRA_N_ worked in a pathogen‐dependent manner. In brief, the successful application of MRRA_L_/MRRA_N_ in reversing the drug resistance of NDM‐producing bacteria and pan‐drug‐resistant *A. baumannii* implied that macromolecule‐based MRRA_S_ possessed broad‐spectrum antimicrobial activity, which was capable to reduce the MIC_MEM_ to the clinically sensitive ranges for carbapenem‐resistant bacteria.

### 2.4 Exploration the Work Mechanisms of MRRA_L_/MRRA_N,_ Revitalizing the Bioactivity of MEM

**FIGURE 3:**
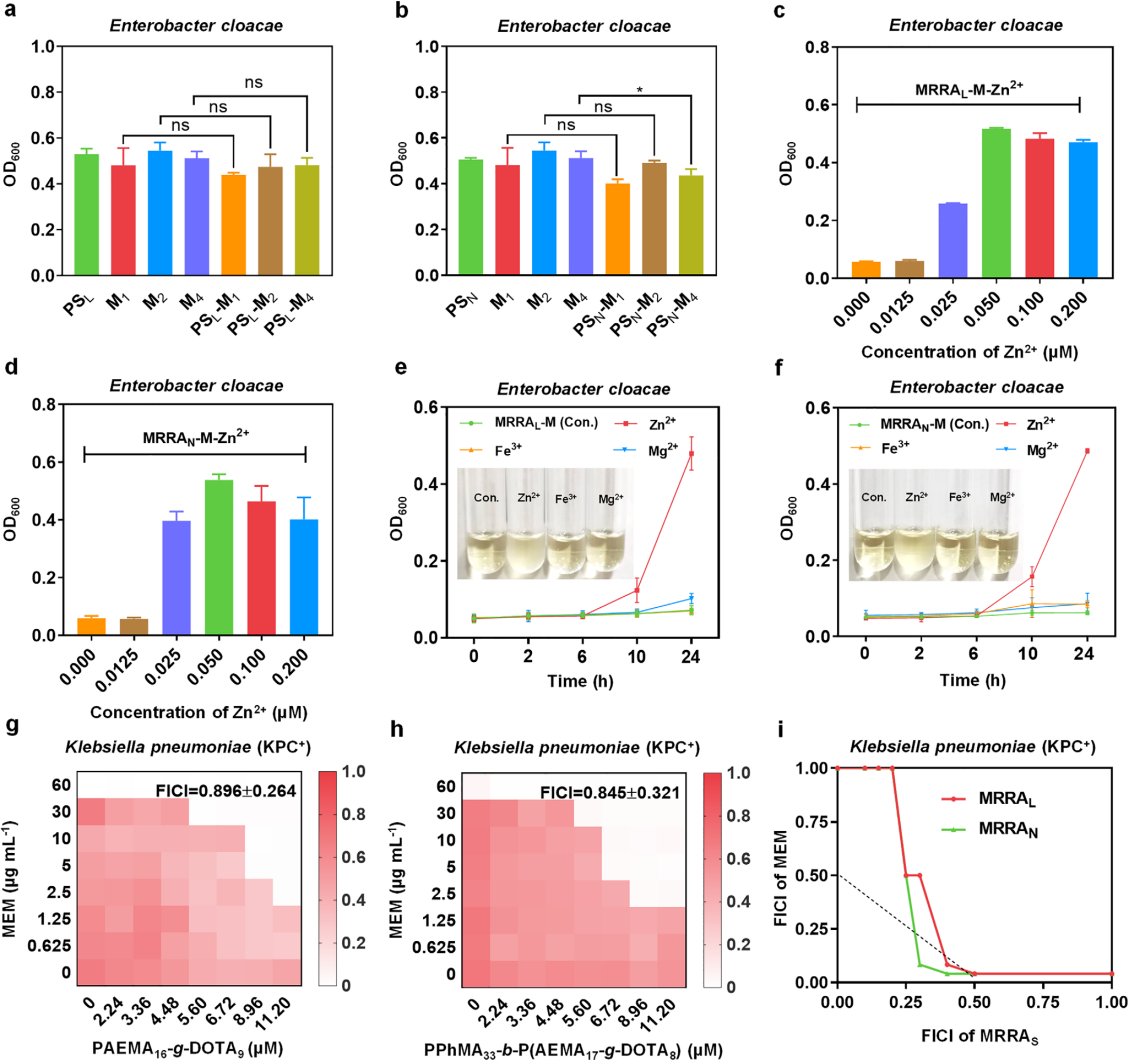
(a, b) The OD_600_ of bacteria treated with different concentrations of MEM (1 µg mL^−1^ (M_1_), 2 µg mL^−1^ (M_2_), and 4 µg mL^−1^ (M_4_)) with or without PS_L_ (5.60 µm) / PS_N_ (5.60 µm of PPhMA_33_‐*b*‐PAEMA_25_. (c, d) The OD_600_ of bacteria treated by MEM (M, 2 µg mL^−1^) combined with MRRA_L_ (5.60 µm) / MRRA_N_ (5.60 µm of PPhMA_33_‐*b*‐P(AEMA_17_‐*g*‐DOTA_8_) added with or without a series concentration of Zn^2+^ in the medium. (e, f) Time‐dependent growth curves of bacteria incubated with MEM (M, 2 µg mL^−1^) combined with MRRA_L_ (5.60 µm) / MRRA_N_ (5.60 µm of PPhMA_33_‐*b*‐P(AEMA_17_‐*g*‐DOTA_8_)) in the medium added with varieties of metal ions (Zn^2+^, Mg^2+^, Fe^3+^) at the concentration of 0.05 µm. (g, h) The checkerboard assays to determine synergism between MEM and MRRA_L_/MRRA_N_ against KPC‐producing *K. pneumoniae*. (i) Isobolograms of the co‐therapy of MEM combined with MRRA_L_/MRRA_N_ against KPC‐producing *K. pneumoniae*. The triangle areas represent the synergistic area. Data are presented as mean ± SD (*n* = 3); ns stands for nonsignificant and ^*^ indicates *p* < 0.1. respectively.

In view of the excellent efficacy of MRRA_L_/MRRA_N_ potentiating the bioactivity of MEM, we sought to clarify how did them work. At first, the efficacy of the polycationic scaffold PS_L_/PS_N_ (which has no DOTA) was evaluated against NDM‐producing *E. cloacae*. PS_L_ (5.60 µm) / PS_N_ (self‐assembled by 5.60 µm of PPhMA_33_‐*b*‐PAEMA_25_) was selected to be combined with 1, 2, and 4 µg mL^−1^ of MEM (shorted as M_1_, M_2_, M_4_) fighting against the bacteria, respectively. After 24 h co‐incubation, there were barely OD_600_ values falling in the co‐therapy groups, keeping nearly the same growth status with monotherapy groups, which made quite a difference from the efficacy of MRRA_L_/MRRA_N_ (Figure 3a,b), demonstrating that the polycationic scaffolds had limited MEM potentiating ability. So, it could be inferred that the grafted DOTA plays a major role in restoring the susceptibility of MEM, just as expected. Given that Zn^2+^ is crucial for NDM, we try to verify that MRRA_L_/MRRA_N_ restores MEM activity by depriving it of Zn^2+^ behaving NDM‐inhibition function through pharmacophore DOTA incorporated within the molecular structure. Subsequently, the Zn^2+^ supplementation experiment was conducted according to a previous report [34]. In brief, a series of diluted Zn^2+^ solutions was added in to the medium containing MEM and MRRA_L_/MRRA_N_. After 24 h of coincubation, the OD_600_ was recorded. No‐added of Zn^2+^ group was taken as control. As shown in the Figure 3c,d, in the added groups, the inhibited bacteria regained the growth ability with the increased concentration of Zn^2+^, while the control group maintained a sustained inhibition state, implying that MRRA_L_/MRRA_N_ mainly functioned through the limitation of Zn^2+^. Furthermore, other metal ions (like Mg^2+^, Fe^3+^) were unable to restore bacterial growth ability under the same concentration of Zn^2+^ added (Figure 3e,f), demonstrating the superior affinity between MRRA_L_/MRRA_N_ and Zn^2+^ compared with other metal ions, that was beneficial to improve the selectivity of MRRA_S_ for therapy in vivo. To sum up, MRRA_L_/MRRA_N_ performing the NDM inhibition function is in close correlation with the Zn^2+^ limiting acquisition. MRRA_L_/MRRA_N_ can “take away” Zn^2+^ from the active center of NDM and the surrounding environment through “DOTA‐like” metal chelating, cutting off the Zn^2+^ supply, inactivating or even structurally disintegrating NDM. When the bacteria were incubated in Zn^2+^‐rich environment, which is sufficient to compensate for the depletion of Zn^2+^, MRRA_L_/MRRA_N_ will lose the ability to reverse the drug resistance of NDM‐producing bacteria.

Finally, a Zn^2+^‐independent drug‐resistant strain‐*Klebsiella pneumoniae* carbapenemase (KPC)‐producing *Klebsiella pneumoniae* (*K. pneumoniae*) was used for further validation for the underlying inhibitory mechanisms of MRRA_L_/MRRA_N_. KPC‐producing *K. pneumoniae* was identified by the same way as other NDM‐producing *Enterobacteriaceae* (Figures S10 and S16). The KPC is one of the carbapenemases belonging to class A of the ambler classification, which possesses a serine residue as an active center, which shares a very different catalytic mechanism from NDM. [38, 39] The checkerboard assays showed that there was hardly synergism between MEM and MRRA_L_/MRRA_N_ fighting against KPC‐producing *K. pneumoniae*. As shown in Figure 3g–i, MRRA_L_/MRRA_N_ had limited MEM potentiating effects even at 11.2 µm of polymers, with the FICIs of 0.896 ± 0.264 and 0.845 ± 0.321, respectively, indicating that MRRA_L_/MRRA_N_ could not effectively reverse the resistance of KPC‐producing *K. pneumoniae*, for which the resistant mechanism is not related to Zn^2+^. In conclusion, MRRA_L_/MRRA_N_ reverse the drug resistance of NDM‐producing bacteria mainly depending on inactivating NDM by depriving the dynamically dissociating Zn^2+^ in the active center of NDM through the incorporated pharmacophore DOTA inside of the molecular structure.

### 2.5 Investigating the Nano‐enhancement Effect of MRRA_N_

**FIGURE 4:**
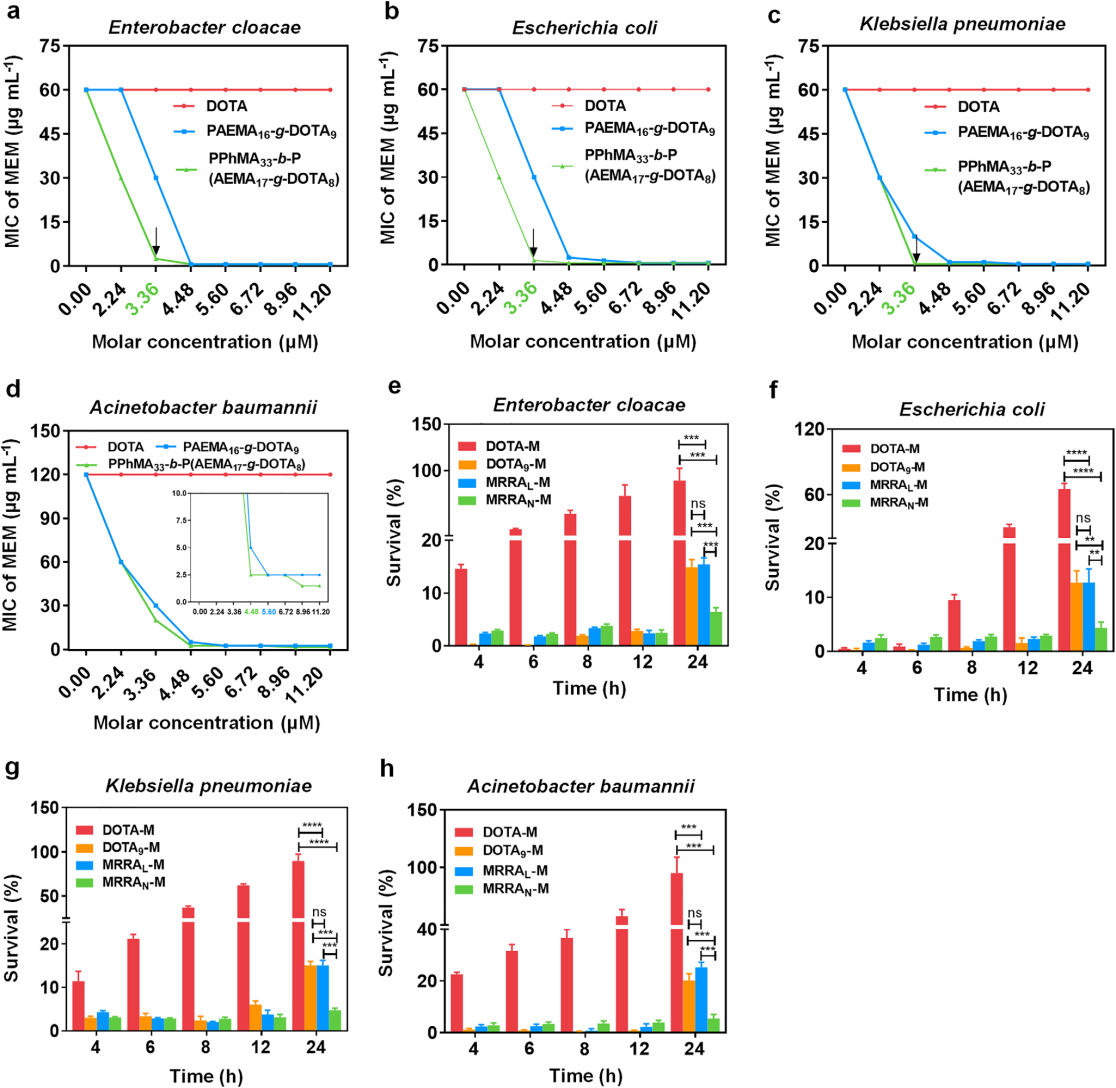
Investigating the nano‐enhancement effect of MRRA_N_. (a–d) The MIC_MEM_ of strains (NDM‐producing *E. cloacae*, NDM‐producing *E. coli*, NDM‐producing *K. pneumoniae*, and pan‐drug‐resistant *A. baumannii*.) after combined with a series of concentration gradients of free DOTA, PAEMA_16_‐*g*‐DOTA_9_ (MRRA_L_) and PPhMA_33_‐*b*‐P(AEMA_17_‐*g*‐DOTA_8_), which functioned in a nano form as MRRA_N_ by self‐ assembly. (e–h) Bacterial survival rate in the co‐therapy groups of MEM combined with free DOTA, MRRA_L_, MRRA_N_, and “equivalent DOTA” (DOTA_9_), shorted as DOTA‐M, MRRA_L_‐M, MRRA_N_‐M, and DOTA_9_‐M for NDM‐producing *E. cloacae*, NDM‐producing *E. coli*, NDM‐producing *K. pneumoniae*, and pan‐drug‐resistant *A. baumannii*. Data are presented as mean ± SD (*n* = 3); ns stands for nonsignificant. ^**^, ^***^, ^****^ indicate *p* < 0.01, *p* < 0.001, and *p* < 0.0001, respectively.

According to the above‐mentioned conclusions, “DOTA motif” was vital for the activity of MRRA_L_/MRRA_N_, we attempted to gain a deeper insight on the superiority of macromolecules due to the multivalent effect, especially the nano‐reinforcement activity, and verified by 4 strains of NDM‐producing *E. cloacae*, NDM‐producing *E. coli*, NDM‐producing *K. pneumoniae*, and pan‐drug‐resistant *A. baumannii*. First, the MIC_MEM_ for all above strains were recorded after incubated with a series of concentration gradients of free DOTA, PAEMA_16_‐*g*‐DOTA_9_ (MRRA_L_), and PPhMA_33_‐*b*‐P(AEMA_17_‐*g*‐DOTA_8_, which functioned in a nano form as MRRA_N_ by self‐ assembly. The minimum detection limit of MIC_MEM_ was set at 0.0625 µg mL^−1^. As shown in Figure 4a–d, MRRA_N_ self‐ assembled by 3.36 µµ of PPhMA_33_‐*b*‐P(AEMA_17_‐*g*‐DOTA_8_) could dramatically reduce MIC_MEM_ from 60 µg mL^−1^ to nearly the lowest limit against NDM‐producing *E. cloacae*, NDM‐producing *E. coli*, and NDM‐producing *K. pneumoniae*, while MRRA_L_ could only reduce the MIC_MEM_ to 10–30 µg mL^−1^, under the same concentration of polymer (3.36 µµ). Even for the seriously drug‐resistant *A. baumannii*, MRRA_N_ self‐assembled by 4.48 µM of PPhMA_33_‐*b*‐P(AEMA_17_‐*g*‐DOTA_8_) could reduce MIC_MEM_ from 120 to 2.5 µg mL^−1^, whereas at least 5.60 µm of MRRA_L_ was required to achieve the comparable effect. Notably, there were no MIC_MEM_ reduction observed from all the range of tested concentration (0.00–11.20 µµ) in the DOTA group for all the 4 above bacteria, revealing that MRRA_L_/MRRA_N_ has a much higher ability to enhance the bioactivity of MEM than free DOTA, which was contributed to increased local density of pharmacophore DOTA inside the macromolecular structure. Notably, MRRA_N_ was seemed to be more superior than MRRA_L_ under the same concentration of the active group of “DOTA.” In order to validate this observation, subsequently, we investigated the co‐therapy effects of MEM combined with free DOTA (5.6 µm), MRRA_L_ (5.6 µm), and MRRA_N_ (5.6 µm of PPhMA_33_‐*b*‐P(AEMA_17_‐*g*‐DOTA_8_)) fighting against all the above 4 strains. By the way, the “equivalent DOTA” was also be evaluated. According to the ^1^H NMR characterizations, a single polymer of PAEMA_25_ or PPhMA_33_‐*b*‐PAEMA_25_ was grafted with 8–9 molecules of small molecule DOTA. So, we chose 9 times of MRRA**_S_** as “equivalent DOTA” concentration, which was denoted by DOTA_9_. As the result showed, there were the highest bacterial survival rates in the DOTA‐M (DOTA combined with MEM) group, indicating that free DOTA (5.60 µm) barely intensifies the efficacy of MEM. While along with the increase of doses of DOTA, DOTA_9_ (50.40 µm) showed effective synergism with MEM, which had a comparable effect with MRRA_L_ (5.60 µm). But both DOTA_9_ and MRRA_L_ were inferior to the MRRA_N_ (5.6 µm of PPhMA_33_‐*b*‐P(AEMA_17_‐*g*‐DOTA_8_)) in the later phase of incubation. (Figure 4e–h) At the beginning of the first 12 h, the groups of DOTA_9_‐MEM, MRRA_L_‐MEM, and MRRA_N_‐MEM had almost uniformly effective bacteria inhibition, with the bacterial survival rates maintaining less than 6%. However, the bacteria in both of DOTA_9_‐MEM and MRRA_L_‐MEM groups gradually resumed growth ability, and the bacterial survival rate could reach about 20% after 24 h, while the MRRA_N_‐MEM group remained continuously efficient inhibition for all the 4 strains during the whole observation. In summary, MRRA_L_ or MRRA_N_ behaved at least an 8‐fold higher synergistic effect than free DOTA. And MRRA_N_ showed more efficient and longer‐lasting activity than its linear counterpart MRRA_L_ to reverse the resistance of NDM‐producing bacteria and pan‐drug‐resistant *A. baumannii* due to the nano‐reinforcement effect.

### 2.6 Membrane Compromise of MRRA_N_ due to the Formation of Nanostructure

**FIGURE 5:**
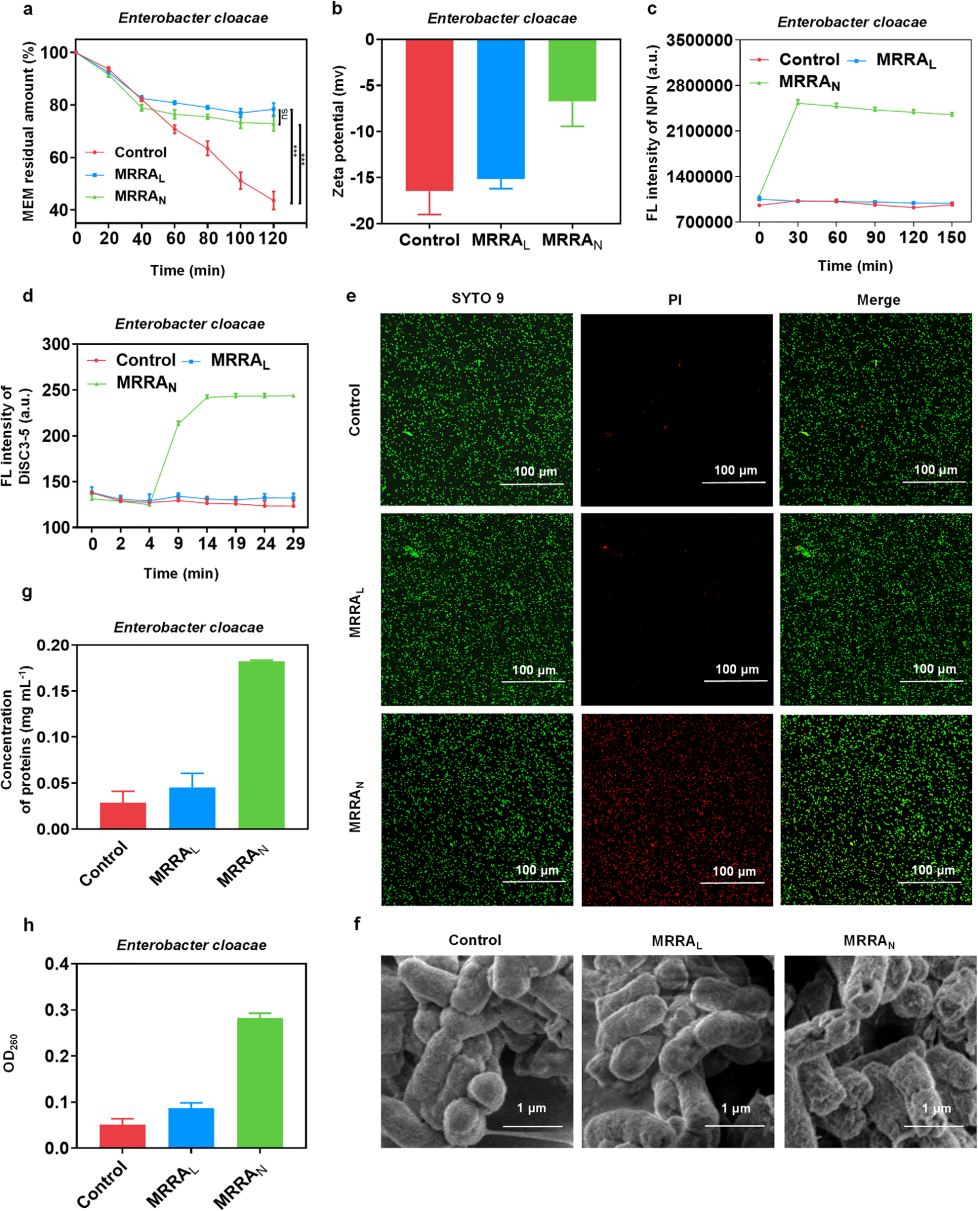
Investigation of the membrane damage effect of MRRA_N_ fighting against NDM‐producing bacteria, except for NDM inhibition. (a) The assay of MEM residue amount after co‐incubation with bacteria pretreated with or without MRRA_L_/MRRA_N_. (b) The Zeta potentials of bacterial surface after treated with or without MRRA_L_/MRRA_N_ for 30 min at the final polymer concentration of 5.60 µm. (c) NPN fluorescent intensity after treated with or without MRRA_L_/MRRA_N_. (d) DiSC3‐5 fluorescent intensity after treated with or without MRRA_L_/MRRA_N_. (e) Bacterial Live/Dead staining after treated with or without MRRA_L_/MRRA_N_, scale bar = 100 µm. (f) Scanning electron microscope of bacteria after treated with or without MRRA_L_/MRRA_N_, scale bar = 1 µm. (g) The protein level after treated with or without MRRA_L_/MRRA_N_. (h) The nucleic acid level after being treated with or without MRRA_L_/MRRA_N_. Data are presented as mean ± SD (*n* = 3); ns stands for nonsignificant. ^***^, indicate, *p* < 0.001.

In order to further clarify the nano‐reinforcement effect, we tried to figure out how did MRRA_N_ outperform its linear counterpart MRRA_L_ from a different perspective under the same conditions of the active group of “DOTA.” The NDM‐producing *E. cloacae* was used to be tested. First, the residual amount of MEM was investigated, which could directly reflect the capacity of NDM inhibition by MRRA_L_/MRRA_N_. After co‐incubated with bacteria pretreated with or without MRRA_L_/MRRA_N_, the amount of MEM was evaluated at the different time points according to a previous report [34]. As shown in Figure 5a, when MEM was co‐incubated with bacteria pre‐treated with MRRA_L_/MRRA_N_ for 120 min, there was around 80% of MEM retained, whereas only about 40% of MEM was preserved for the un‐pre‐treated group, indicating that MRRA_L_/MRRA_N_ could protect MEM from hydrolysis. But there was no significant discrepancy of MEM residue amount between the MRRA_L_ and MRRA_N_ groups, suggesting that MRRA_L_ and MRRA_N_ have a comparable capability to inactivate the NDM. Thus, there must be some kind of other mechanisms to explain the superiority of MRRA_N_.

Subsequently, the membrane‐compromising effect of MRRA_N_ was investigated. Owing to the formation of nano‐micelle, there must be a strong concentration of cationic charge outside of the MRRA_N_, which could compromise the membranes of bacteria effectively through electrostatic interaction and the hydrophobic force inside of the nano‐micelle structure [40, 41]. To verify our speculation, bacteria adhesion assay was performed. After co‐incubated with MRRA_L_/MRRA_N_ for 30 min, unadhered MRRA_L_/MRRA_N_ was removed by centrifugation, the Zeta potentials of bacteria surface were measured. For the MRRA_N_ group, the Zeta potential elevated from around ‐16.47 to ‐6.72 mV, whereas the MRRA_L_ group had weak Zeta potential variation (to about ‐15.17 mV), demonstrating the much stronger bacterial adhesion ability of MRRA_N_ by electrostatic force (Figure 5b). Next, two fluorescent probes were used to determine the permeability of the bacterial outer membrane and depolarization of the plasma membrane. N‐phenyl‐1‐naphthylamine (NPN) is a typical probe for evaluation outer membrane damage, which has intense fluoresces under hydrophobic conditions [42, 43]. In addition, membrane potential‐sensitive fluorescent dye 3,3‐dipropylthiodicarbonyl cyanine iodide (DiSC3‐5) was used to monitor depolarization of bacterial plasma membrane. When the cytoplasmic membrane is perturbed, or the permeability is increased, lipophilic molecules will bind tightly to DiSC3‐5, and result fluorescence increasement [44, 45]. As shown in Figure 5c,d, the obviously elevated NPN and DiSC3‐5 fluorescence were detected in the MRRA_N_ group, while the MRRA_L_ group presented nearly identical fluorescence intensity with the control group, after the bacteria were treated by MRRA_L_ (5.60 µm) / MRRA_N_ (5.60 µm of PPhMA_33_‐*b*‐P(AEMA_17_‐*g*‐DOTA_8_)), respectively. The results demonstrated that MRRA_N_ had much higher membrane damaging property than its linear counterpart, MRRA_L_. Although the membrane‐damaging activity of MRRA_N_ at low polymer concentration (5.60 µm) is not enough to kill the bacteria completely, it would interfere with bacterial metabolic imbalance and weaken their ability to resist the harsh osmolarity environment, resulting in bacteria in a more “weakened” state, which is conducive to boosting the bioactivity of MEM.

Subsequently, bacterial Live/Dead staining and scanning electron microscopy (SEM) were conducted to characterize bacterial membrane damage. SYTO 9 could enter all the bacteria, including integrated cells and damaged cells, then stain the bacteria green; whereas PI could only pass through damaged cells and bind to the nuclear DNA, emitting red fluoresces, which is commonly used to indicate the membrane damage [42, 46]. After stained by the two dyes, the bacteria treated with MRRA_N_ exhibited much stronger red fluorescence than the MRRA_L_ group at the concentration of 5.60 µm of polymers (Figure 5e). What's more, SEM images also displayed that the bacteria in the MRRA_N_ group showed more damaged morphology with rough and wrinkled surface, while the bacteria were relatively smooth and intact in the MRRA_L_ groups, which were characterized nearly the same with the control group (Figure 5f). Furthermore, we also detected leakage of the bacterial constituents (nucleic acids and proteins), which was an important indicator of compromising membrane integrity [37, 47]. The OD_260_ could reflect the content of nucleic acids and the Pyrogallol Red‐molybdate method (PRM) was used to detect the protein content [48, 49]. After treated by MRRA_N_, the nucleic acids and proteins were significantly higher than both of the MRRA_L_ and control groups (Figure 5g,h). Collectively, all of the experiments above confirmed that MRRA_N_ (5.60 µm of PPhMA_33_‐*b*‐P(AEMA_17_‐*g*‐DOTA_8_)) could physically damage bacterial membrane relying on the strong local positive charge and amphiphilic interaction compared to its linear counterpart. The membrane‐damaging property of MRRA_N_ not only interferes with the bacterial metabolism but also greatly facilitates the in‐flow of the nanoparticles and MEM, which maximizes the synergistic effect, leading to better bactericidal activity [50]. After crossing the outer membrane of the bacteria, MRRA_N_ could inhibit NDM located in the cell periplasm by enriched local DOTA concentration. Then, damage the intracellular membrane, leading to the leakage of nucleic acids and proteins. As a result, MRRA_N_ behaved excellent synergistic effects with MEM through multiple mechanism reversing the drug resistance of NDM‐producing bacteria. However, MRRA_L_ may be much more free‐flowing and flexible, which could only access to the cell periplasm to inhibit NDM with a single mechanism.

### 2.7 Hemolysis and the Biocompatibility of MRRA_L_ and MRRA_N_

**FIGURE 6:**
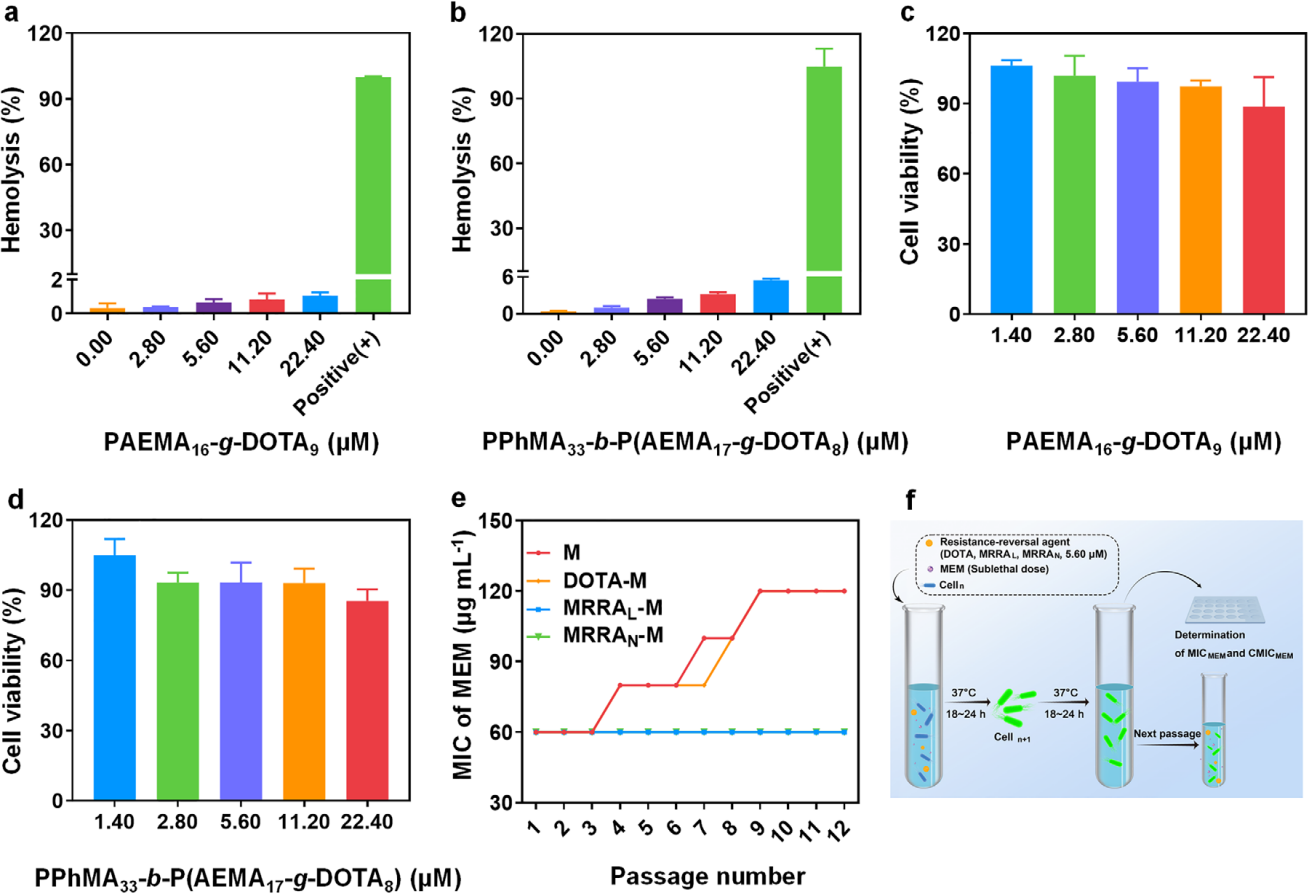
Biosafety and drug‐resistant induction for both MRRA_L_ and MRRA_N_. (a, b) Hemolysis assay treated by different concentration of MRRA_L_ and MRRA_N_. (c, d) The cytotoxicity assay treated by different concentrations of MRRA_L_ and MRRA_N_ for the mouse fibroblast cell line L929 by CCK‐8 assay. (e) The MIC_MEM_ of bacteria induced by MEM alone (M), MEM combined with DOTA (DOTA‐M), MEM combined with MRRA_L_ (MRRA_L_‐M), and MEM combined with MRRA_N_ (MRRA_N_‐M). **f)** Schematic diagram of co‐induction for drug‐resistance by sublethal MEM with or without free DOTA (5.60 µm), MRRA_L_ (5.60 µm) and MRRA_N_ (5.60 µm of PPhMA_33_‐*b*‐P(AEMA_17_‐*g*‐DOTA_8_)).

Given the excellent efficacy of MRRA_L_/MRRA_N_ reversing the drug resistance of NDM‐producing bacteria, good biocompatibility is an important prerequisite for their clinical transformation. The hemolysis experiment was first conducted with red blood cells (RBCs) as previously reported [46, 51]. There was no obvious hemolysis reactions observed for both MRRA_L_ and MRRA_N_ at the polymer concentration ranging from 2.80 to 22.4 µm. (Figure 6a,b) Notably, at the effective antimicrobial concentration (5.6 µm of polymers), the hemolysis rates were almost negligible (around 2%). After that, the cytotoxicity of MRRA_L_ and MRRA_N_ for the mouse fibroblast cell line L929 was evaluated by CCK‐8 assay. As the results showed, the semi‐inhibitory concentration IC_50_ (the concentration of test drug required for 50% cytotoxicity for mouse fibroblasts) of polymers for both MRRA_L_ and MRRA_N_ were significantly higher than 22.4 µm. And negligible cytotoxicity (∼95% of the L929 cells survived) were viewed at the effective antimicrobial concentration (5.60 µm of polymers) (Figure 6c,d). It is evident that both MRRA_L_ and MRRA_N_ are safe for further in vivo application and have the potential to treat refractory infections caused by NDM‐producing bacteria.

### 2.8 Development for Drug Resistance

As an effective approach to combat drug resistance, co‐therapy not only improves the efficacy and reduces the toxicity of existing antibiotics, but also minimizes the tendency for drug resistance evolution [52]. Subsequently, we investigated the capability of MRRA_L_/MRRA_N_ in mitigating the drug resistance onset. In addition, the long‐term effect of MRRA_L_/MRRA_N_ should be further assessed. Because with continuous stimulation of MRRA_L_/MRRA_N_, bacteria will probably develop more advanced metalloenzymes to compete with Zn^2+^ with MRRA_L_/MRRA_N_ or develop other resistance mechanisms, such as deletion of pore protein, increased efflux pumps, etc., to resist the harsh environment, [50, 53] resulting in in‐effectivity of MRRA_L_/MRRA_N_. Briefly, the bacteria were incubated in the broth containing sub‐MIC_MEM_ with free DOTA (5.60 µm), MRRA_L_ (5.60 µm), and MRRA_N_ (5.60 µm of PPhMA_33_‐*b*‐P(AEMA_17_‐*g*‐DOTA_8_)). Then MIC_MEM_ for each subpopulation of bacteria in all the groups was monitored. After 12 passages, the MIC_MEM_ in both MEM and DOTA‐M groups increased from the original 60 to 120 µg mL^−1^, while the MIC_MEM_ in the groups of MRRA_L_‐M and MRRA_N_‐M remained unchanged (Figure 6e), implying that MRRA_L_ and MRRA_N_ could mitigate the development of drug resistance, but free DOTA didn't work. The schematic for the co‐induction of MEM with free DOTA, MRRA_L,_ and MRRA_N_ was shown in Figure 6f. The dose of MEM which was needed to be combined with of MRRA_L_ (5.60 µm) / MRRA_N_ (5.60 µm of PPhMA_33_‐*b*‐P(AEMA_17_‐*g*‐DOTA_8_)) to suppress the bacteria efficiently was annotated as CMIC_MEM_, which was monitored for every passage of the subpopulation. The CMIC_MEM_ in the MRRA_L_‐M group has raised from the beginning of 1 to 2 µg mL^−1^ after 6 passages, while in MRRA_N_‐M group, the CMIC_MEM_ was increased from 0.5 to 1 µg mL^−1^ after 8 passages, then both the CMIC_MEM_ in the two groups remained unchanged until the end of observation (Table S2). These results demonstrated that low dose of MRRA_L_ (5.60 µm) / MRRA_N_ (5.60 µm of PPhMA_33_‐*b*‐P(AEMA_17_‐*g*‐DOTA_8_)) could still be effective to reverse the drug resistance of NDM‐producing bacteria, reducing the MIC_MEM_ of bacteria to a clinically sensitive range despite long‐term exposure. In brief, MRRA_L_ and MRRA_N_ are two potent adjuvants with high‐efficient and long‐term activity to boost effectivity of MEM with less propensity for drug resistance to fight against NDM‐producing bacteria.

### 2.9 Therapeutic Performance of MRRA_L_‐M /MRRA_N_‐M in Pneumonia Treatment

**FIGURE 7:**
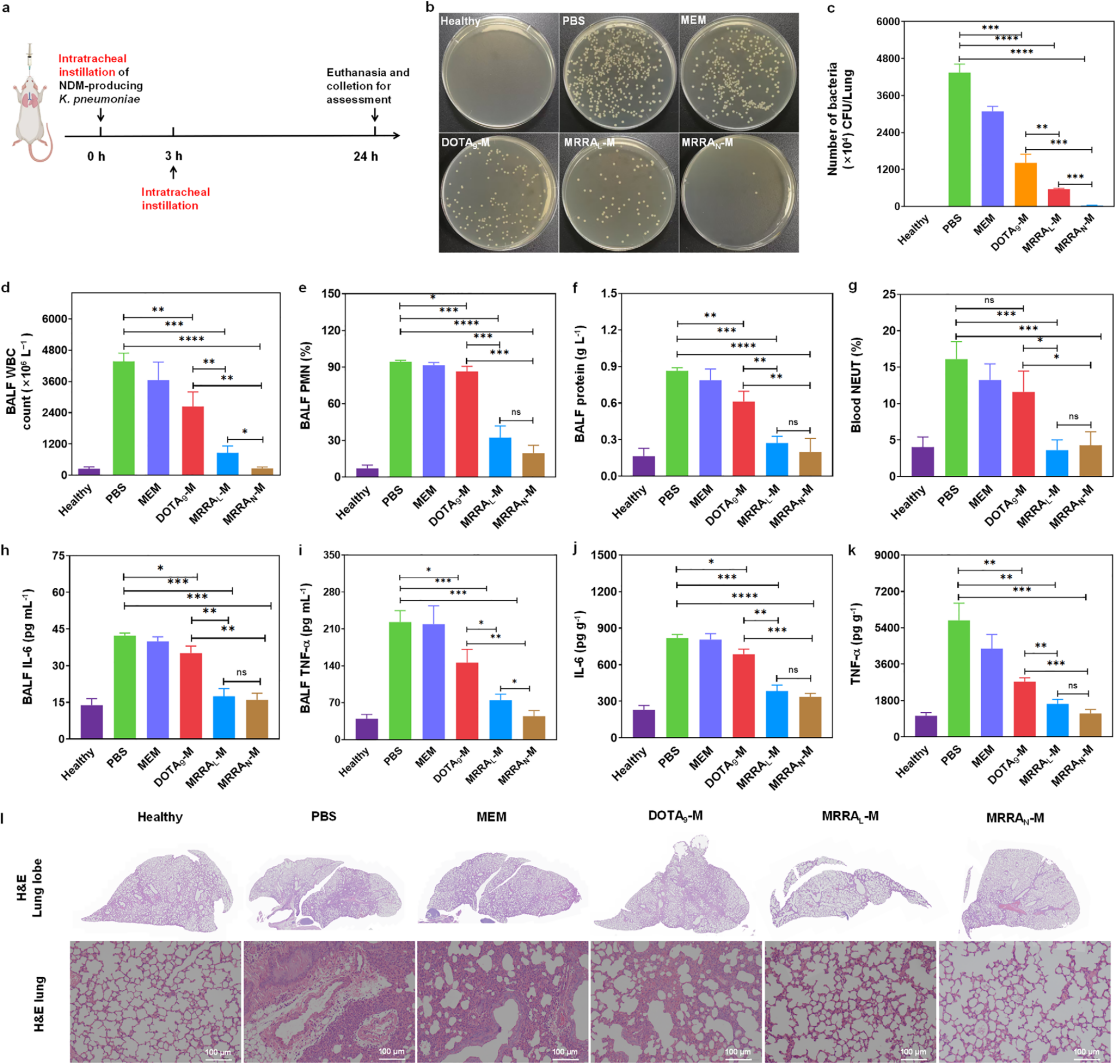
The therapeutic efficacy evaluation for the treatment of acute pneumonia caused by NDM‐producing *K. pneumonia* via intratracheal instillation. (a) Schematic illustration for modeling and treatment. (b, c) The bacterial loads with different therapeutic processes. (d–g) The levels of WBC (d), PMN% (e), total protein content in BALF (f), and NEUT% (g) in blood from different treatments. (h, i) The levels of IL‐6 and TNF‐α in BALF from different treatments. (j, k) The levels of IL‐6 and TNF‐α in lung tissue homogenate supernatant from different treatments. (l) H&E staining images of lung from different treatment groups. Data are presented as mean ± SD (*n* = 5); ns stands for nonsignificant. ^*^, ^**^, ^***^, ^****^ indicate *p* < 0.05, *p* < 0.01, *p* < 0.001, and *p* < 0.0001, respectively.

Given the clinical challenge of pneumonia caused by NDM‐producing *K. pneumoniae—*a condition frequently requiring combination therapy with highly toxic antibiotics such as tigecycline and polymyxin, which can lead to severe hepatorenal dysfunction and neurotoxicity [54, 55]. We established a murine acute pneumonia model via intratracheal instillation of NDM‐producing *K. pneumoniae* (5 × 10^8^ CFU mL^−^^1^, 50 µL). Three hours post‐infection, mice were treated with PBS, MEM, adjuvant monotherapy (DOTA_9_, MRRA_L_, or MRRA_N_), or combination therapy (DOTA_9_‐M, MRRA_L_‐M, or MRRA_N_‐M) administered intratracheally. Bronchoalveolar lavage fluid (BALF), blood, and lung tissues were collected 24 h post‐infection to evaluate the in vivo therapeutic efficacy of MRRA_L_‐M and MRRA_N_‐M (Figure 7a).

Colony counting of lung tissue homogenates revealed the highest bacterial load in the PBS group, confirming successful model establishment (Figure 7b,c). Compared with all monotherapy groups (MEM or adjuvants alone) and the DOTA_9_‐M combination, both MRRA_L_‐M and MRRA_N_‐M significantly reduced bacterial loads, demonstrating potent in vivo antibacterial activity (Figure 7b,c and Figure S17). Notably, although DOTA_9_‐M showed antibacterial activity comparable to MRRA_L_‐M in vitro, the in vivo performance was inferior. This discrepancy underscores the advantages of macromolecular therapeutics, which benefit from prolonged circulation and sustained action patter. Moreover, at an equivalent “DOTA” active group dose (5.04 µmol kg^−^^1^), MRRA_N_‐M reduced bacterial loads by at least one order of magnitude more than MRRA_L_‐M. This enhanced efficacy may be attributed not only to nano‐reinforced antibacterial activity but also to higher local concentrations facilitated by nanoscale size. Overall, these results indicate that MRRA_L_ and MRRA_N_ hold strong potential for clinical translation in combatting NDM‐producing bacterial resistance, offering a novel polycationic macromolecular adjuvant strategy against severe drug‐resistant infections.

Consistent with the reduction in bacterial loads, MRRA_L_‐M and MRRA_N_‐M combination therapy also significantly decreased white blood cell counts (WBC) (Figure 7d), polymorphonuclear leukocyte (PMN) percentage (PMN%) (Figure 7e), and total protein content in BALF (Figure 7f), as well as blood neutrophil (NEUT) percentage (NEUT%) (Figure 7g). These improvements were not observed in monotherapy groups or the DOTA_9_‐M combination group (Figure S18). Similarly, levels of proinflammatory cytokines (IL‐6, TNF‐α) in both BALF and lung tissue homogenate supernatant were markedly reduced following MRRA_L_‐M and MRRA_N_‐M treatment, but not or slightly reduced in the groups of monotherapy groups or the DOTA_9_‐M combination group (Figure 7h–k and Figure S19; standard curves shown in Figure S20). Together, these data indicate that MRRA_L_‐M and MRRA_N_‐M effectively attenuate lung inflammation and promote functional recovery.

What's more, hematoxylin and eosin (H&E) staining of lung tissues was acquired for evaluating the therapeutic effect, which revealed extensive hemorrhage, edema, and a hardened texture in PBS, monotherapy, and DOTA_9_‐M combination groups, reflecting severe tissue damage and inflammation secondary to high bacterial loads. In contrast, lungs from MRRA_L_‐M and MRRA_N_‐M treatment groups remained bright red and soft, with minimal histological injury, showing the best tissue repair, with near‐normal alveolar architecture (Figure 7l and Figure S21). These findings confirm that the in vitro efficacy of MRRA_L_‐M and MRRA_N_‐M translates effectively in vivo, providing a promising therapeutic option against refractory NDM‐producing bacterial infections, for which clinical treatments remain limited.

Toxicity was also evaluated via H&E staining and serum biochemical markers. After treatment, blood and major organs (heart, liver, spleen, and kidney) were collected. No significant differences were observed in serum levels of alanine transaminase (ALT), aspartate transaminase (AST), blood urea nitrogen (BUN), or creatinine (SCR) between healthy mice and those treated with MRRA_L_‐M or MRRA_N_‐M (Figure S22). H&E staining of major organs revealed no notable structural abnormalities or inflammatory infiltration in the combination therapy groups of MRRA_L_‐M and MRRA_N_‐M, resembling findings in healthy controls (Figure S23). These results support the good biocompatibility and safety profile of MRRA_L_‐M and MRRA_N_‐M for in vivo application.

## 3 Conclusion

In this work, we engineered two macromolecular resistance‐reversal agents (MRRA_S_): MRRA_L_ and MRRA_N_, both of them exhibited powerful and long‐term activity to restore efficacy of MEM in vivo, without the development of drug resistance. Notably, this antibacterial effect can be effectively translated into in vivo therapy, significantly reducing lung damage caused by NDM‐producing *K. pneumoniae*. MRRA_L_ and MRRA_N_ could compete the Zn^2+^ in the activity pocket of NDM, relying on the incorporated “DOTA” inside the polycationic scaffold, leading to NDM inactivation, and preventing the antibiotics from hydrolysis. Particularly, MRRA_N_ is more promising due to its formation of nanostructures. MRRA_N_ not only possesses efficient NDM inhibition ability through the multivalent effect of pharmacophore “DOTA” around the bacteria, but also physically damages bacterial membranes owing to the intensified local positive charge and hydrophobic force, which is beneficial for the uptake of both MEM and MRRA_N,_ maximizing the synergistic efficacy. In addition, as particles with a nano‐size, MRRA_N_ could target the site of lesion by EPR effects and selectively attacks on the negatively charged bacterial surfaces other than mammalian cells (usually neutral charged), which is helpful to improve the efficacy and biosafety in vivo. In conclusion, compared with traditional small molecular NDM inhibitors, MRRA_L_/MRRA_N_, especially MRRA_N,_ with multiple antibacterial mechanisms, provide a novel approach for overcoming the drug resistance of NDM‐producing bacteria, combining with the advantage of “macromolecule,” “polycation,” and “Zn^2+^ competition.” More importantly, the strategy of pharmacophore immigration provides a practicable attempt to engineer more MRRA_S_ based on polycation with other active small molecules to fight against the resistance of NDM‐producing bacteria.

## 4 Experimental Section

### 4.1 Bacterial Strains and Materials

NDM‐producing *E. cloacae*, NDM‐producing *E. coli*, NDM‐producing *K. pneumoniae*, KPC‐producing *K. pneumoniae*, and pan‐drug‐resistant *A. baumannii* were isolated by the clinical laboratory of Shanxi Bethune Hospital. Luria–Bertani broth (LB), Cation‐Adjusted Mueller‐Hinton broth (CAMHB), and Tryptone Soya broth (TSB) were purchased from Guangdong Huankai Biotechnology Co., Ltd. EDTA (0.5 mm) solution was purchased from Guangzhou Yubo Biotechnology Co., Ltd. Meropenem (MEM) and N‐phenyl‐1‐naphthylamine (NPN) were purchased from Shanghai Yuanye Biotechnology Co., Ltd. Xpert Carba‐R Assay was obtained from Cepheid (USA). 1,4,7,10‐Tetraazacyclododecane‐N,N,N'',N'''‐tetraaceticacid (DOTA) and 3,3‐dipropylthiodicarbonyl cyanine iodide (DiSC3‐5) were purchased from Shanghai Jizhi Biochemical Technology Co., Ltd. 1,4,7,10‐Tetraazacyclododecane‐1,4,7,10‐tetraacetic acid mono‐N‐hydroxysuccinimide ester (DOTA‐NHS) was purchased from Xi'an Haoran Biotechnology Co., Ltd. Dimethyl sulfoxide (DMSO) was purchased from Tianjin Jindong Tianzheng Fine Chemical Reagent Factory. 4‐(benzenecarbonothioylsulfanyl)‐4‐cyanopentanoic acid (CTA) was purchased from Bidepharm medicine (Shanghai, China). 2‐Aminoethyl methacrylate hydrochloride (AEMA) and Azobisisobutyronitrile (AIBN) were obtained from Shanghai Aladdin Biochemical Technology Co., Ltd. Triethylamine (Et_3_N) was purchased from Shandong Xiya Chemical Industry Co., Ltd. Ethanol, methanol, ethylacetate, dichloromethane, and petroleum ether were obtained from Tianjin Beichen Fangzheng Reagent Company. Trifluoroacetic acid, 2‐phenoxyethyl methacrylate (PhMA), ZnCl_2_, Fe (NO_3_)_3,_ and MgCl_2_ were purchased from Shanghai Macklin Biochemical Co., Ltd. SYTO 9 was purchased from Thermo Fisher Scientific (USA). Propidium iodide (PI) staining solution and Cell Counting Kit (CCK‐8) were obtained from Shanghai Yeasen Biotechnology Co., Ltd. Tert‐butanol was purchased from Tianjin Damao Chemical Reagent Factory. DMEM (high glucose), fetal bovine serum (FBS), dual antibiotics (penicillin, streptomycin), and trypsin were all purchased from Beijing Seven Biotech Co., Ltd. Mouse TNF‐α ELISA Kit and Mouse IL‐6 ELISA Kit were purchased from Dogesce (Beijing, China). Water used in this study was deionized with a Milli‐Q reagent water system.

### 4.2 Screening and Confirmation of Strains

Modified carbapenem inactivation method (mCIM) and EDTA‐carbapenem inactivation method (eCIM) were performed to identify the carbapenem‐resistant *Enterobacteriaceae* strains and to preliminarily distinguish the enzyme types: metalloenzyme or serine carbapenemase, according to CLSI M100‐S32. Briefly, 1 loop (1 µL) of the bacteria colony was picked from the overnight culture plates, then added into 2 mL of TSB with or without 20 µL of 0.5 m EDTA (the test without EDTA called mCIM and the test added with EDTA called eCIM), followed by placing the discs containing 10 µg of MEM into the TSB broth incubating for 4 h at 35°C in an atmospheric environment. Subsequently, removed out the discs and attached them to MHA plates that had been pre‐coated with *Escherichia coli* ATCC25922, and continued incubating for 18–24 h at 35°C. Then, the inhibition zones were measured. Interpretation of the results: 1) mCIM (‐): inhibition zone ≥ 19 mm, indicating carbapenemase negative, with the result of eCIM invalid. 2) mCIM (+) and eCIM (‐): both of the inhibition zones = 6 mm, indicating serine carbapenemase positive. 3) mCIM (+) and eCIM (+): inhibition zone of mCIM = 6 mm and inhibition zone of eCIM ≥ 15 mm, indicating metalloenzyme positive. Subsequently, the candidate strains were detected on the GeneXpert Infinity‐48s (Cepheid, USA) to confirm the carbapenem resistance gene. Eventually, 1 NDM‐producing *E. cloacae*, 1 NDM‐producing *E. coli*, 1 NDM‐producing *K. pneumoniae*, and 1 KPC‐producing *K. pneumoniae* were verified for the experiments. In addition, 1 pan‐drug‐resistant *A. baumannii* also was isolated, which was severely resistant to all the clinically available antibiotics, including carbapenems. All strains were inoculated in 20% glycerol broth and stored at ‐80°C for next use.

### 4.3 Synthesis of PAEMA_25_ (PS_L_)

PAEMA_25_ was synthesized via the RAFT polymerization method. Briefly, 266.76 mg (1.61 mmol) of AEMA, 30.00 mg (0.107 mmol) of CTA, and 3.54 mg (0.021 mmol) of AIBN were dissolved in DMSO (1.2 mL) into a schlenk tube. The tube was degassed via three freeze‐pump‐thaw cycles and sealed under vacuum, then reacted in an oil bath at 70°C for 24 h. The polymerization was terminated in liquid nitrogen, and then precipitated into excessive ethylacetate to obtain a pink product. The residue was completely dissolved by methanol and then reprecipitated into excessive ethylacetate again. After repeating 3 times, the residue was dried overnight in a vacuum oven. ^1^H NMR and GPC were used to characterize the product.

### 4.4 Synthesis of PPhMA_33_‐b‐PAEMA_25_

PAEMA_25_ was further employed as a macro‐RAFT agent for the polymerization. Typically, PAEMA_25_ (100 mg, 0.022 mmol), PhMA (145 mg, 0.703 mmol), and AIBN (0.7757 mg, 0.0047 mmol) were dissolved in DMSO (1.2 mL) into a schlenk tube. The tube was degassed via three freeze‐pump‐thaw cycles and sealed under vacuum, then reacted in an oil bath at 70°C for 24 h. After 24 h, the polymerization was terminated in liquid nitrogen, and then precipitated into methyl tert‐butyl ether and centrifugate. After dissolved the residue in dichloromethane and precipitated it with a large amount of petroleumether, repeated this process three times. Finally, the residue was dried overnight in a vacuum oven. ^1^H NMR and GPC were used to characterize the product.

### 4.5 Synthesis of MRRA_L_ and MRRA_N_

DOTA‐NHS ester was grafted with PAEMA_25_ to synthesize MRRA_L_. In brief, PAEMA_25_ (20 mg, 0.0045 mmol), DOTA‐NHS ester (34 mg, 0.067 mmol) and Et_3_N (10.3 mg, 0.101 mmol) were dissolved in DMSO (1 mL) into a schlenk tube. The tube was sealed under vacuum, then reacted at room temperature for 36 h. After that, the mixture was purified in a dialysis bag with MWCO = 3500 KDa to dialyze against deionized water. Finally, a light pink product of PAEMA_16_‐*g*‐DOTA_9_ (MRRA_L_) was acquired after freeze‐drying. Using the same method, replace PAEMA_25_ (20 mg, 0.0045 mmol) with PPhMA_33_‐*b*‐PAEMA_25_ (30 mg, 0.00267 mmol) to synthesize PPhMA_33_‐*b*‐P(AEMA_17_‐*g*‐DOTA_8_). ^1^H NMR and GPC were used to characterize the product. Subsequently, PPhMA_33_‐*b*‐P(AEMA_17_‐*g*‐DOTA_8_) (1 mg) was dispersed in 1 mL deionized water under ultrasound (240 W, 30 s ON) at first. After that, further ultrasound (450 W, 5 s ON, 5 s OFF) was conducted for 5 min to obtain a colloidal solution of MRRA_N_, which appeared clear and transparent.

### 4.6 Nuclear Magnetic Resonance (NMR) Spectra Characterization of Polymers

In detail, 15 mg of PAEMA and 15 mg of PAEMA‐*g*‐DOTA were dissolved in 600 µL of D_2_O, respectively. 15 mg of PPhMA‐*b*‐PAEMA was dissolved in 600 µL of DMSO‐d6. And 18 mg of PPhMA‐*b*‐P(AEMA‐*g*‐DOTA) was dissolved in 600 µL of methanol‐d4 with a little of trifluoroacetic acid (20 µL). Before detection, make sure all the polymer solution was clear and transparent. All the ^1^H NMR results were obtained on a Bruker AVANCE III 400 MHz NMR spectrometer.

### 4.7 Gel Permeation Chromatography (GPC) Characterization of Polymers

GPC was conducted to analyze the molecular mass distribution of the products. Briefly, 15∼18 mg of polymers were used for assays. The mobile phase for PAEMA, PAEMA‐*g*‐DOTA, PPhMA‐*b*‐PAEMA, and PPhMA‐*b*‐P(AEMA‐*g*‐DOTA) was aqueous phase, aqueous phase, DMSO, and methanol with a bit of trifluoroacetic acid (1:30), respectively. The initial concentration of the sample upon entering the chromatographic column was 0.10 mg mL^−1^, and the injection volume was 100 µL. All the data were obtained on PL‐GPC50 (USA).

### 4.8 Zeta Potential and Hydrodynamic Diameter

300 µg mL^−1^ PSL (PAEMA_25_), MRRA_L_ (PAEMA_16_‐*g*‐DOTA_9_), PS_N_ (self‐assembled by 300 µg mL^−1^ of PPhMA_33_‐*b*‐PAEMA_25_) and MRRA_N_ (self‐assembled by 300 µg mL^−1^ of PPhMA_33_‐*b*‐P(AEMA_17_‐*g*‐DOTA_8_)) were prepared, and then detected Zeta potential and hydrodynamic diameter assay by dynamic light scattering (Malvern, NANO ZS90, U.K).

### 4.9 Transmission Electron Microscope (TEM) for MRRA_N_

Prepare the solution of MRRA_N_ (300 µg mL^−1^) according to the previous method. Followed by dropping 20 µL of the solution onto the 300‐mesh copper grid coated with carbon support film (Zhongjingkeyi Technology Co., Ltd) vertically, repeated 3∼5 times. Air‐dry naturally overnight and detected using a transmission electron microscope (TEM, JEM‐2100F, JEOL Japan) under an accelerating voltage of 200 kV.

### 4.10 Minimum Inhibitory Concentration (MIC)of MEM

The MIC of the MEM assay was performed as previously reported by broth microdilution method [56, 57]. Typically, the bacteria at logarithmic phase were collected at 3000 rpm for 5 min, followed by repeated washing by PBS for 3 times. Then the sediment was resuspended by PBS to the final concentration of 5 ∼ 10 × 10^5^ CFU mL^−1^. MEM was diluted serially with MH broth with the concentration ranging from 240 to 1.25 µg mL^−1^. Then, 100 µL of MEM solution was added to 96‐well plate, co‐incubated with 100 µL of bacteria suspension at 37°C for 18–24 h. The MIC was defined as the lowest concentration of antibiotic with no visible growth of bacteria.

### 4.11 Checkerboard Method

The checkerboard assays were conducted to determine the FICI between MEM and PAEMA_16_‐*g*‐DOTA_9_ (MRRA_L_) / PPhMA_33_‐*b*‐P(AEMA_17_‐*g*‐DOTA_8_) functioned in a nano form as MRRA_N_ according to the previous description with minor modifications [33]. First, both MEM and MRRA_L_/MRRA_N_ were diluted serially with MH broth, then 50 µL of each single solution of MEM and MRRA_L_/MRRA_N_ were added into 96‐well plate along the horizontal and vertical rows respectively to obtain the mixture solution of MEM and MRRA_L_/MRRA_N_. Subsequently, 100 µL of bacteria suspension (5 ∼ 10 × 10^5^ CFU mL^−1^) was added and co‐cultured for 18–24 h at 37°C. Finally, OD_600_ of each well was recorded by a microtiter plate reader (PerkinElmer, USA). FICI was calculated by the following formula as:

(1) $$\mathrm{FICI}=\frac{\mathrm{MI}C_{\left(\mathrm{MEM},\mathrm{combination}\right)}}{\mathrm{MI}C_{\left(\mathrm{MEM},\;\mathrm{alone}\right)}}+\frac{\mathrm{MI}C_{\left(\mathrm{MRR}A_{L}/\mathrm{MRR}A_{N},\;\mathrm{combination}\right)}}{\mathrm{MI}C_{\left(\mathrm{MRR}A_{L}/\mathrm{MRR}A_{N},\mathrm{alone}\right)}}$$

### 4.12 Zn^2+^ Supplementation Experiment

Briefly, 50 µL of MRRA_L_ (22.4 µm) / MRRA_N_ (self‐assembled by 22.4 µm of PPhMA_33_‐*b*‐P(AEMA_17_‐*g*‐DOTA_8_)), 50 µL of MEM (8 µg mL^−1^), and 50 µL of bacterial suspension (5 ∼ 10 × 10^5^ CFU mL^−1^, NDM‐producing E. cloacae) were co‐incubated in a 96‐well plate at 35°C. Then 50 µL of various concentrations of ZnCl_2_ (0.00–0.8 mm) was added into the wells, 24 h later the OD_600_ was recorded. Furthermore, the time‐time‐dependent growth curves by adding 50 µL of ZnCl_2_, Fe (NO_3_)_3_, MgCl_2_ with the final concentration of 0.05 µm were also recorded at the observation points of 0, 2, 6, 10, and 24 h.

### 4.13 Residual MEM Detection

This experiment was performed according to the previous report [34]. In brief, the overnight cultured NDM‐producing E. cloacae was diluted to OD_600_ = 0.05 with LB. Then, MRRA_L_ (5.60 µm) / MRRA_N_ (self‐assembled by 5.60 µm of PPhMA_33_‐*b*‐P(AEMA_17_‐*g*‐DOTA_8_)) was added to the bacteria suspension and incubated at 35°C for 3 h. Bacteria cells were collected by centrifugation and washed by PBS for 3 times, then resuspended the bacteria to OD_600_ = 0.3. Subsequently, 50 µL of the above bacteria suspension was added into a 96‐well plate, and co‐incubated with 50 µL of MEM solution (95.86 µg mL^−1^) at room temperature. The OD_300_ values were monitored by a microtiter plate reader (SYNERGY/H1, Bio Tek, USA) at the observation points of 0, 20, 40, 60, 80, 100, and 120 min, respectively. The blank was set as 50 µL of PBS. The residual MEM was calculated by the following formula as:

(2) $$\mathrm{Residual}\;\mathrm{MEM}\;\mathrm{amount}\left(\%\right)=\frac{OD_{V}-OD_{b}}{OD_{0}-OD_{b}}\times 100\%$$

where OD_0_ represents the absorbance at 0 min. OD_v_ represents the absorbance at other monitored points of 20, 40, 60, 80, 100, and 120 min. OD_b_ represents the observation of a blank well.

### 4.14 Bacterial Zeta Potential Analysis

1 mL of bacteria suspension (5 ∼ 10 × 10^5^ CFU mL^−1^, NDM‐producing E. cloacae) was mixed with MRRA_L_/MRRA_N_ with the final polymer concentration at 5.60 µm, and co‐incubated for 30 min at room temperature. Subsequently, the bacteria cells were collected by centrifuged at 3000 rpm for 5 min, then the sediment was resuspended in 1 mL of PBS for measuring the Zeta potential The bacteria without treatment was used as a control.

### 4.15 Outer Membrane Permeation Assay

Bacterial suspension (NDM‐producing E. cloacae) with the concentration of 5 ∼ 10 × 10^5^ CFU mL^−1^ was prepared according to the above procedure. NPN was dissolved in methanol and diluted with PBS for the following experiment. Next, 100 µL of bacteria suspension, 50 µL of NPN solution (with a final concentration at 10 µm), and 50 µL of MRRA_L_/MRRA_N_ (with a final polymer concentration at 5.60 µm) were mixed into 96‐well plates and co‐incubated at room temperature in the dark. The fluorescence of NPN was monitored by a microtiter plate reader (SYNERGY/H1, Bio Tek, USA) at the observation points of 0, 30, 60, 90, 120, and 150 min (Ex: 350 nm, Em:420 nm). The bacteria treated with sterile water were used as a control.

### 4.16 Cytoplasmic Membrane Depolarization

The overnight cultured NDM‐producing *E. cloacae* was collected at 3000 rpm for 5 min, then washed by buffer A (5 mm HEPES, 20 mm glucose) for 3 times, and resuspended the bacteria in buffer B (5 mm HEPES, 20 mm glucose, 100 mm KCl and 0.2 mm EDTA, pH = 7.4) to the concentration of 5 ∼ 10 × 10^5^ CFU mL^−1^. The DiSC3‐5 was dissolved in DMSO first, and then diluted with PBS to a concentration of 4 µm. Next, 100 µL of bacteria suspension and 50 µL DiSC3‐5 were added into 96‐well plates and incubated at room temperature. The fluorescence of the plate was monitored by a multifunctional reading instrument (Varioskan Flash, Thermo Fisher, USA) (Ex: 622 nm, Em: 690 nm). When the fluorescence intensity remained stable, 50 µL of MRRA_L_/MRRA_N_ was added into the 96‐well plates with the final polymer concentration of 5.60 µm. The fluorescence intensity was monitored every 5 min. The bacteria treated with sterile water were used as a control.

### 4.17 Bacteria LIVE/DEAD Viability Assay

The overnight cultured NDM‐producing E. cloacae was collected and adjusted to the concentration of OD_600_ = 1.0 in 0.85% NaCl for use. 1 mL of bacterial suspension was mixed with MRRA_L_/MRRA_N_ with the final polymer concentration at 11.20 µm and co‐incubated at 35°C for 30 min. Subsequently, bacterial cells were collected and washed by 0.85% NaCl for 3 times. Then, the sediment was suspended into dye solution (0.75 µm of PI and 1.25 µm of SYTO 9) and co‐incubated in the dark at room temperature for 30 min. Then, bacterial cells were centrifugated and washed by 0.85% NaCl to remove unbound dye. After that, the sediment was suspended into 0.85% NaCl and observed by CLSM (FLUOVIEW FV3000, OLYMPUS). The bacteria treated with sterile water were used as a control.

### 4.18 Morphology Observation by Scanning Electron Microscopy (SEM)

In brief, 1 mL of the bacterial suspension (OD_600_ = 1.0) was mixed with MRRA_L_/MRRA_N_ (the final polymer concentration was 11.20 µm), and co‐incubated at 35°C for 30 min. The bacterial cells were centrifugated and washed by PBS for 3 times to remove the unbound materials. Then, bacterial cells were fixed with 2.5% glutaraldehyde at 4°C for 4 h. Next, the samples processed a graded ethanol dehydration procedure (30%, 50%, 70%, 80%, 90% and 100% of ethanol) for 15 min every time, and replaced with tert‐butanol. Finally, the samples were dried by vacuum freeze‐drying and observed via SEM (Carl Zeiss AG Gemini 300). The bacteria treated with PBS were used as a control.

### 4.19 Hemolytic Activity Assay

The potential toxicity of MRRA_L_/MRRA_N_ against red blood cells (RBCs) was evaluated by a hemoglobin release assay. Typically, 500 µL of fresh RBCs were centrifuged at 4000 rpm for 10 min, the supernatant was removed, and RBCs were washed gently with PBS for additional 3 times. The precipitate cells were dispersed into 5 mL of PBS. Subsequently, 300 µL of RBC solution were taken and mixed with serially diluted MRRA_L_/MRRA_N_ with the total volume reaching at 1 mL, then co‐incubated at 35°C for 4 h. After that, each sample was centrifuged at 4000 rpm for 10 min, and the absorbance of the supernatant at 540 nm was examined using a microplate reader (PerkinElmer, USA). The groups treated with PBS and deionized water were used as negative and positive controls, respectively. The hemolysis rate was calculated as:

(3) $$\mathrm{Hemolysis}\;\left(\%\right)=\frac{OD_{0}-OD_{1}}{OD_{2}-OD_{1}}\times 100\%$$

where OD_0_, OD_1_, and OD_2_ represent the absorbance of the experimental, negative, and positive control groups, respectively.

### 4.20 Cell Viability Assays

Cell viability assays were determined by CCK‐8 assay as previously reported [58]. The mouse fibroblast cells (L929) were selected to evaluate the cytotoxicity of MRRA_L_/MRRA_N_. Briefly, approximately 1 × 10^4^ cells cultured with DMEM supplemented with 10% FBS and 1% antibiotics (100 µg mL^−1^ streptomycin and 100 U penicillin) were seeded into a 96‐well microplate. After 24 h incubation (35°C, 5% CO_2_), the medium of each well was removed gently, and serially diluted MRRA_L_/MRRA_N_ were added into each well and co‐incubated for another 24 h (35°C, 5% CO_2_). The cell viability was measured by the CCK‐8 assay according to the manufacturer's instructions, and calculated using the following formula as:

(4) $$\mathrm{Cell}\;\mathrm{Viability}\left(\%\right)=\frac{OD_{0}-OD_{1}}{OD_{2}-OD_{1}}\times 100\%$$

where OD_0_, OD_1_, and OD_2_ represent the absorbances of the experimental, blank, and control groups, respectively.

### 4.21 Induction of Drug Resistance

Briefly, the bacteria of NDM‐producing *E. cloacae* were induced in the medium containing sub‐MIC of MEM with or without DOTA (5.60 µm), MRRA_L_ (5.60 µm), and MRRA_N_ (self‐assembled by 5.60 µm of PPhMA_33_‐*b*‐P(AEMA_17_‐*g*‐DOTA_8_). After every passage, each evolved bacteria was harvested in fresh LB medium for another 24 h incubation at 35°C to obtain stable bacteria cells for evaluating of the evolved MIC_MEM_ and CMIC_MEM_ combined with DOTA (5.60 µm), MRRA_L_ (5.60 µm), and MRRA_N_ (self‐assembled by 5.60 µm of PPhMA_33_‐*b*‐P(AEMA_17_‐*g*‐DOTA_8_)) as well as for the next passage. By following the same steps, 12 passages were repeated.

### 4.22 Establishment of the Mice Pneumonia Model

All animal experiments were performed in accordance with the Guidelines for Institutional Animal Care and Use Committee and approved by the Ethics Committee of Shanxi Provincial People's Hospital (Approval no.2024‐868). A total of 8–9 weeks‐old (weighed ≈20 g), female BALB/c mice were purchased from Beijing Viton Lever Company. Mice rooms have a 12‐h dark‐light cycle, the room temperature is around 24°C, and the humidity is kept at 45–65%. And each mouse was normal in appearance, diet, daily routine, and behavioral habits before the experiment. Briefly, NDM‐producing *K. pneumoniae* was cultured overnight, and diluted to OD_600_ = 0.5 (5 ∼ 10 × 10^8^) with 0.85% NaCl for use. The mouse was anesthetized, intratracheally instilled of 50 µL of the bacterial suspension, and the vertical position was maintained for 3 min to ensure that all *K. pneumoniae* entered the lungs.

### 4.23 Therapeutic Evaluation

The infected mice were randomly divided into groups (n = 15) and administered with different drug formulas after 3 h post infection. In detail, 50 µL of PBS, MEM (100 µg kg^−1^), DOTA_9_ (2.038 mg kg^−1^), MRRA_L_ (4.984 mg kg^−1^), MRRA_N_ (8.624 mg kg^−1^ of PPhMA_33_‐b‐P(AEMA_17_‐g‐DOTA_8_)) single drug and DOTA_9_ (2.038 mg kg^−1^)‐M (100 µg kg^−1^), MRRA_L_ (4.984 mg kg^−1^)‐ M (100 µg kg^−1^), MRRA_N_ (8.624 mg kg^−1^ of PPhMA_33_‐b‐P(AEMA_17_‐g‐DOTA_8_))‐M (100 µg kg^−1^) dual‐drug were directly delivered to the lung by the airway. After 24 h, the mice were sacrificed, and their tissues (including heart, liver, spleen, lung and kidney), BALF, and blood were collected for efficacy and safety evaluation. The bacterial loads of lung were counted by agar plating. For the histological study, the tissues were fixed by 10% paraformaldehyde for H&E staining. TNF‐α and IL‐6 factors were checked by the ELISA method, which showed the condition of inflammation. The WBC and PMN% in BALF, as well as NEUT% in blood, were detected by Sysmex XN‐2800. The total protein in BALF was checked by the pyrogallol colorimetric method. The ALT, AST, BUN, and SCR were conducted on Beckman Coulter AU5800.

### 4.24 Statistical Analysis

Each experiment was performed at least three times. Results are presented as means ± SD. Statistical analysis was performed using GraphPad Prism 8.0.1 software with the method of unpaired T test (two‐sided testing), ^*^ *p* < 0.05, ^**^ *p* < 0.01, ^***^ *p* < 0.001, and ^****^ *p* < 0.0001 were considered statistically significant.

## Conflicts of Interest

The authors declare no conflicts of interest.

## Supporting information




**Supporting File**: advs74696‐sup‐0001‐SuppMat.docx.

## Data Availability

Data supporting the findings of this study are available from the corresponding author upon request.
