# Supplementary material for: DOTA‐grafted Cationic Polymers Behaving as Powerful Macromolecular Resistance‐reversal Agents (MRRAs) Combating Against New Delhi Metallo‐β‐lactamase (NDM)‐producing Bacteria
Source: Adv Sci (Weinh). 2026 Mar 5;13(27):e19056. doi: 10.1002/advs.202519056 (PMC13170275; doi:10.1002/advs.202519056)
Supplement: Supplementary file 1 — Supporting File: advs74696‐sup‐0001‐SuppMat.docx. [file ADVS-13-e19056-s001.docx]

Supporting Information

**DOTA-grafted cationic polymers behaving as powerful macromolecular resistance-reversal agents (MRRAs) combating against New Delhi metallo-β-lactamase (NDM)-producing bacteria**

*Ruixue Wang* *^a, b^* *^#^, Jian Zhang* *^c #^, Yun li* *^a^* *^#^, Liping Qiao* *^a^, Zhuorui Dong* *^b^, Dandan Cui* *^b^, Peirong Bai* *^a^, Liping Li* *^b,^* *^d^* *^*^, Bing Cao* *^a^ ^*^, and Ruiping Zhang ^b^* *^*^*

1. Shanxi Bethune Hospital, Shanxi Academy of Medical Sciences, Third Hospital of Shanxi Medical University, Tongji Shanxi Hospital, Taiyuan, 030032, China
2. The Radiology Department of Shanxi Provincial People’s Hospital, Five Hospital of Shanxi Medical University, Taiyuan, 030001, China
3. Key Laboratory of Interface Science and Engineering in Advanced Materials Ministry of Education, Taiyuan University of Technology, Taiyuan, 030024, China
4. School of Basic Medical Sciences, Shanxi Medical University, Taiyuan, 030001, China

^#^ Ruixue Wang, Jian Zhang, Yun Li contributed equally to the study.

*Corresponding:* *liliping@sxmu.edu.cn; caobing@sxbqeh.com.cn; zrp_7142@sxmu.edu.cn*


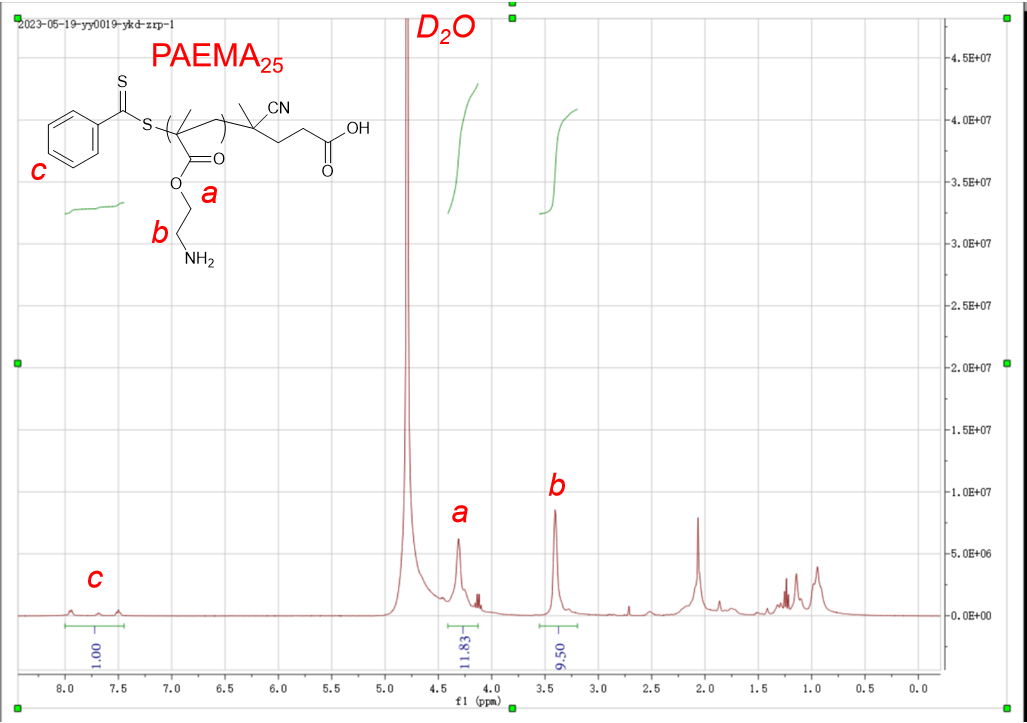


**Figure S1.** ^1^H NMR spectrum recorded for polycation PAEMA_25_ in D_2_O.


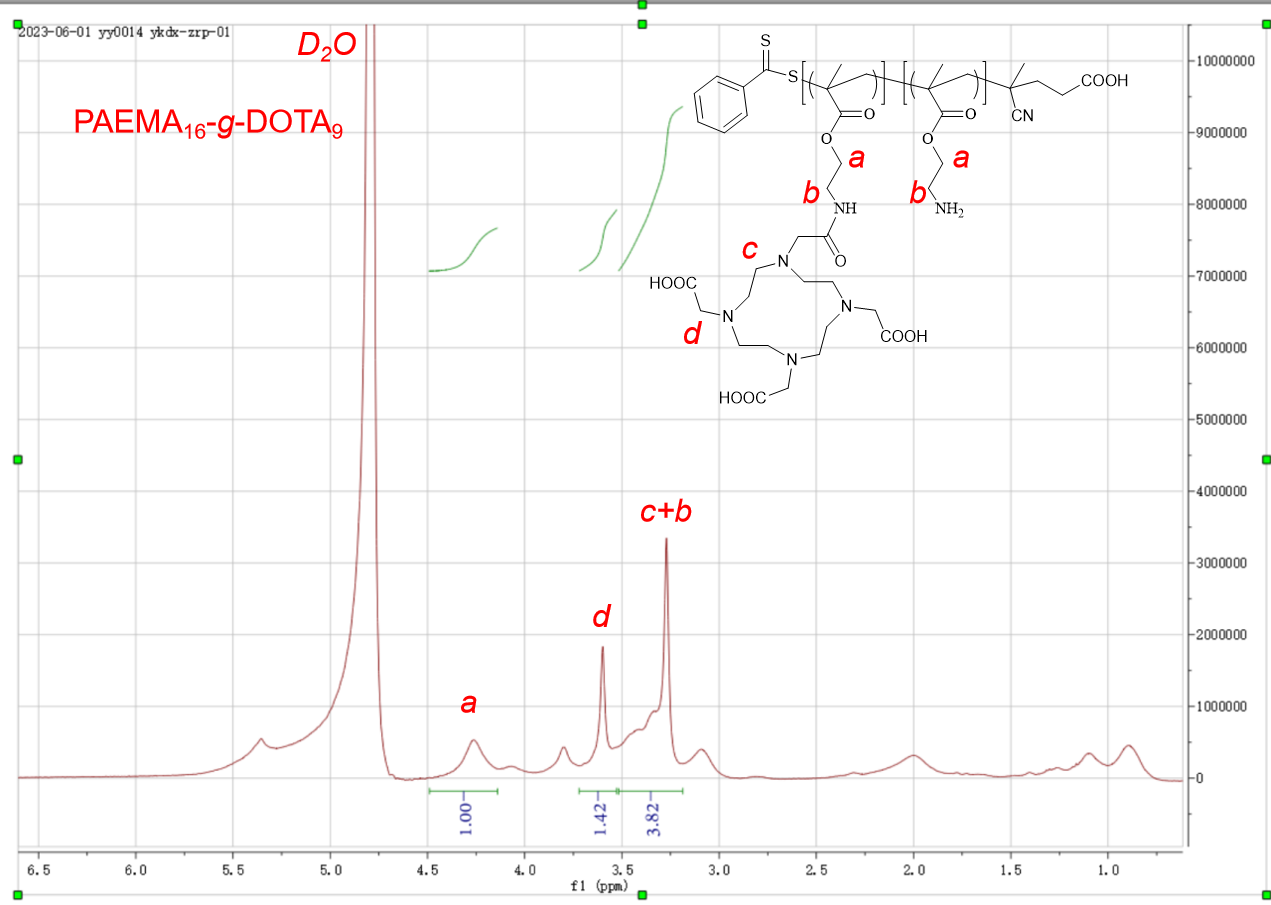


**Figure S2.** ^1^H NMR spectrum recorded for PAEMA_16_-*g*-DOTA_9_ in D_2_O.


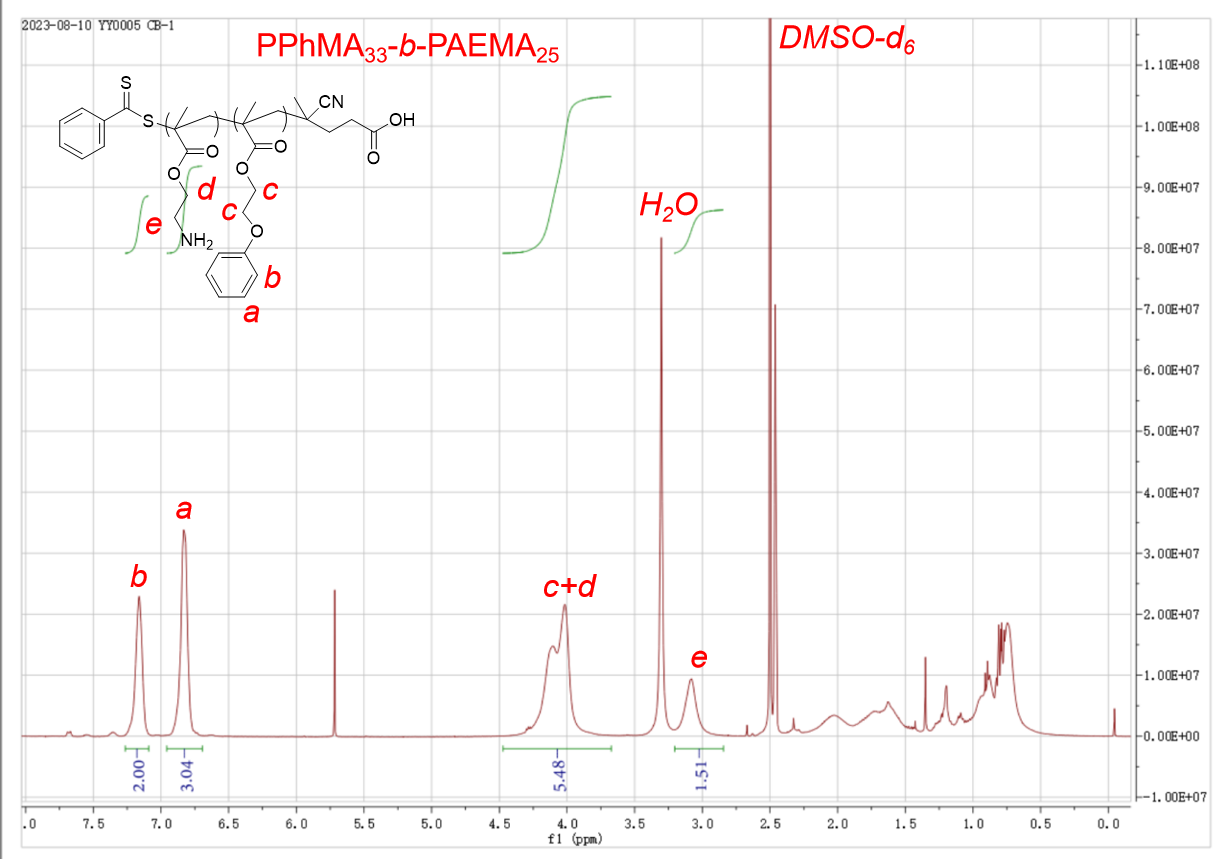


**Figure S3.** ^1^H NMR analysis of the amphiphilic polycation PPhMA_33_-*b*-AEMA_25_ in DMSO-d_6_.


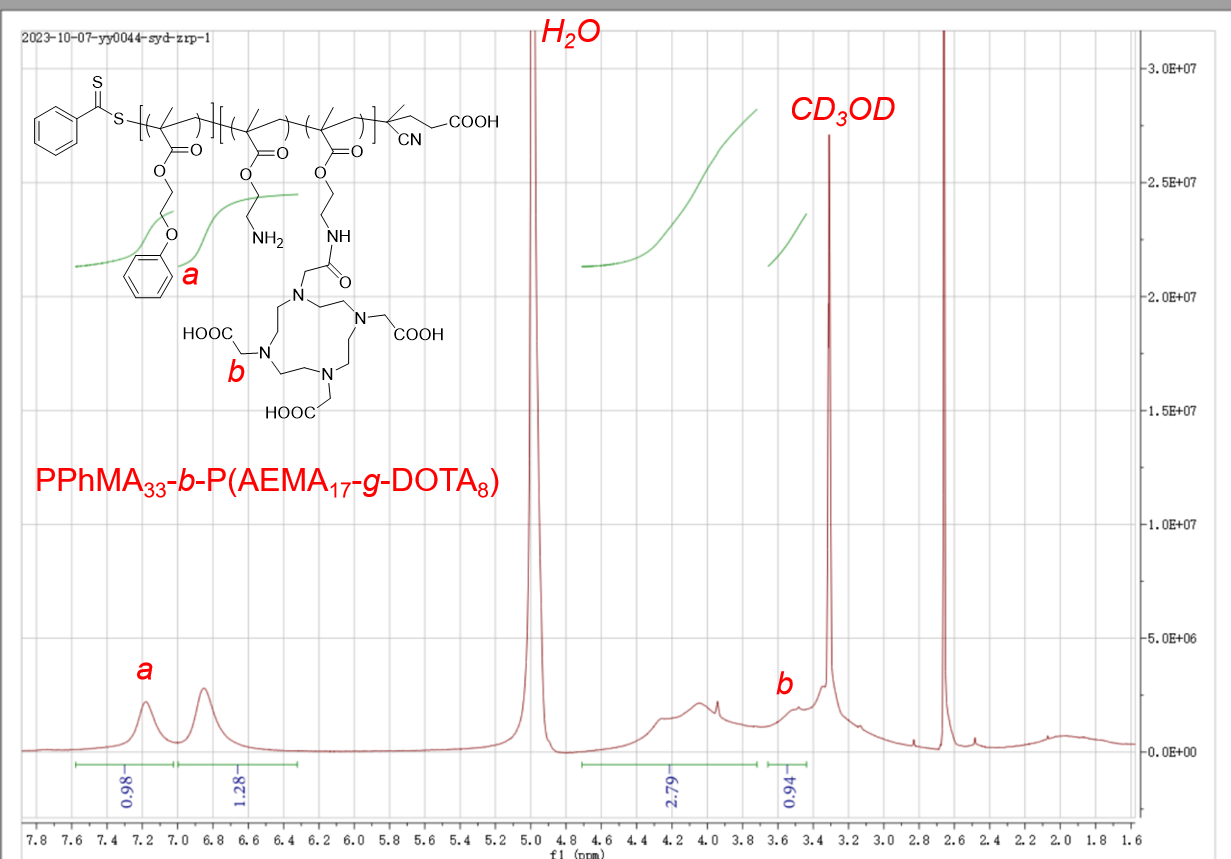


**Figure S4.** ^1^H NMR analysis of the amphiphilic polymer PPhMA_33_-*b*-P(AEMA_17_-*g*-DOTA_8_)in CD_3_OD.


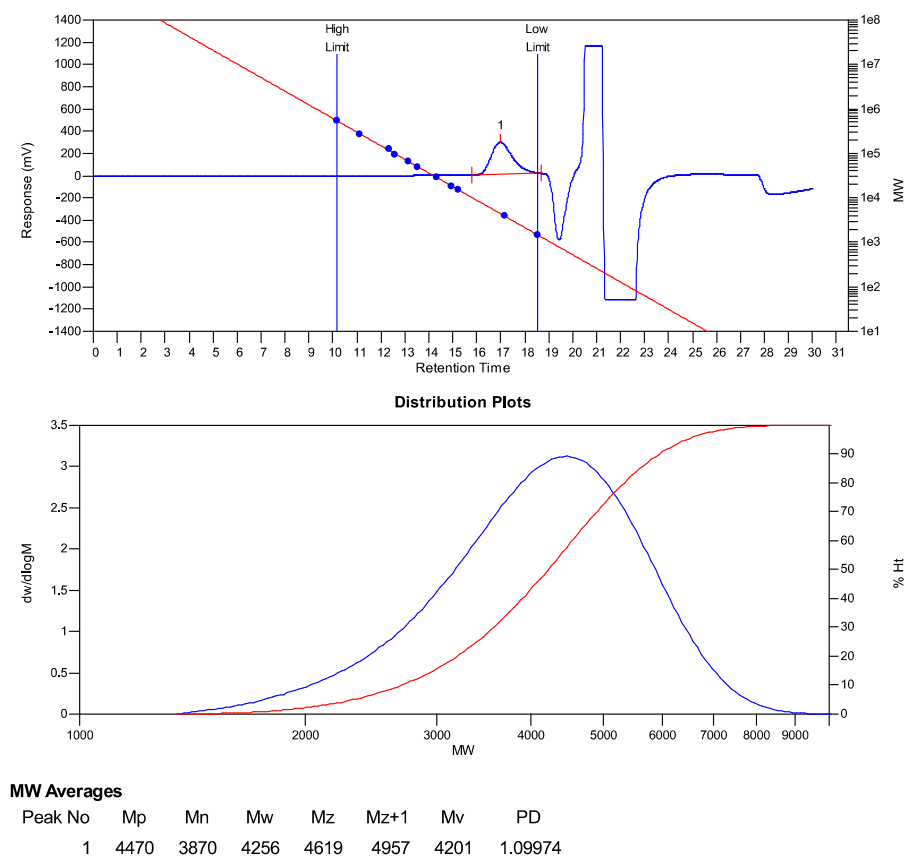


**Figure S5.** The GPC result for polycation of PAEMA_25_.


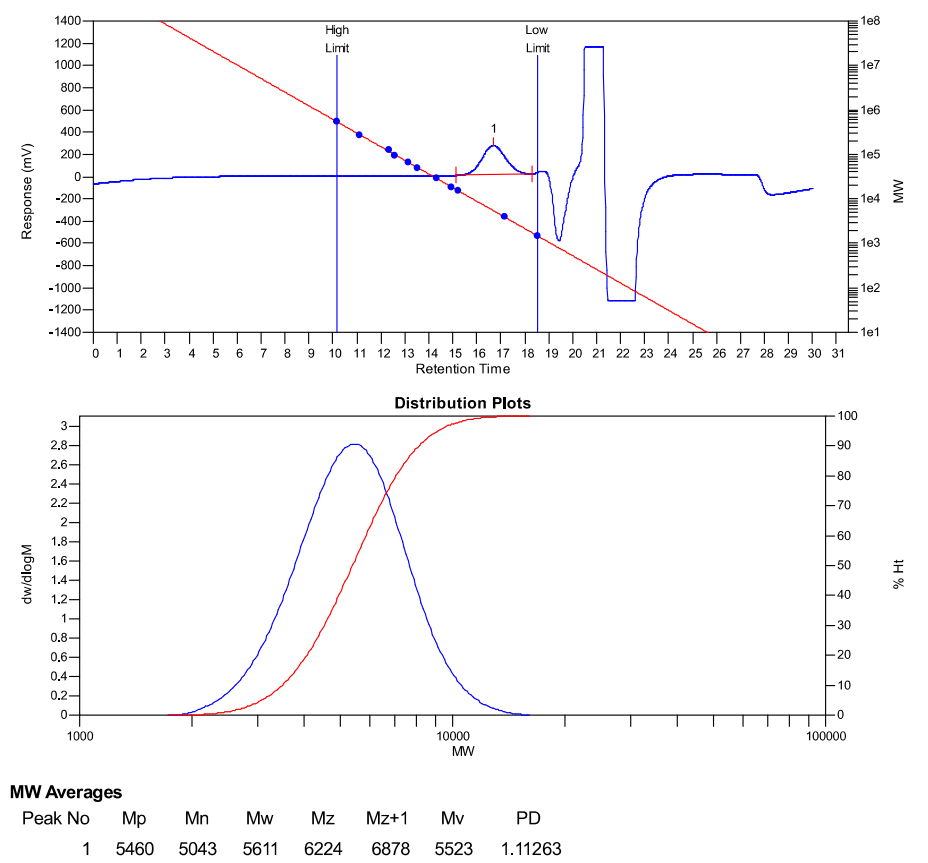


**Figure S6.** The GPC result for PAEMA_16_-*g*-DOTA_9_.


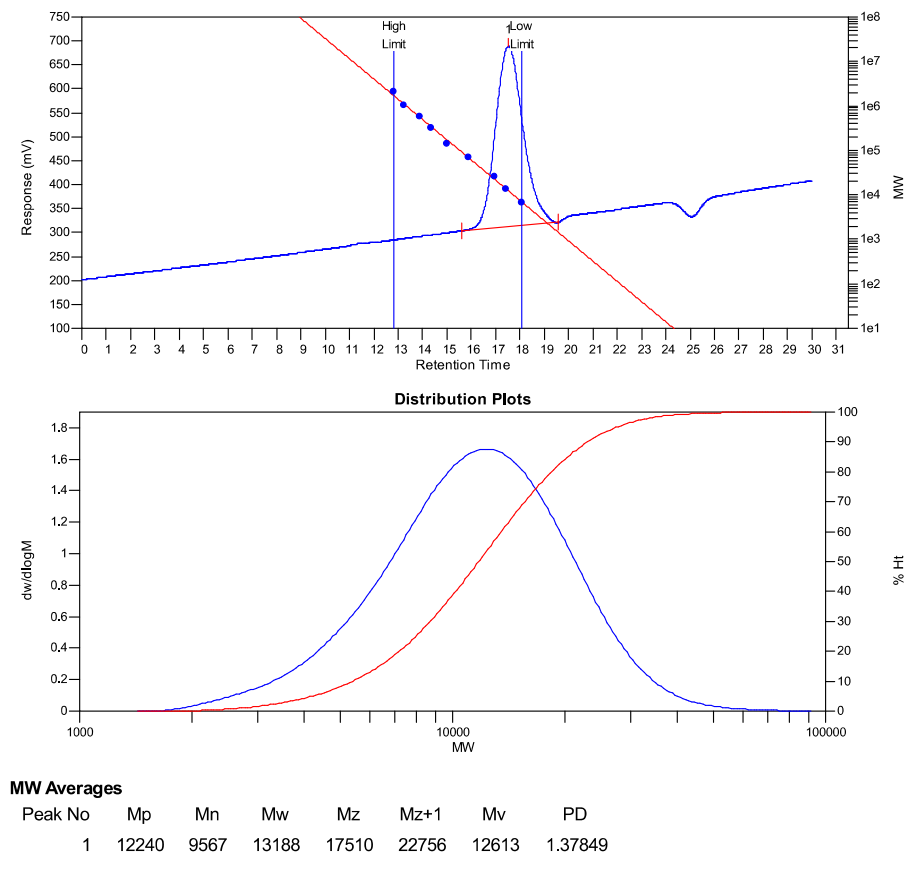


**Figure S7**. The GPC result for PPhMA_33_-*b*-PAEMA_25_.


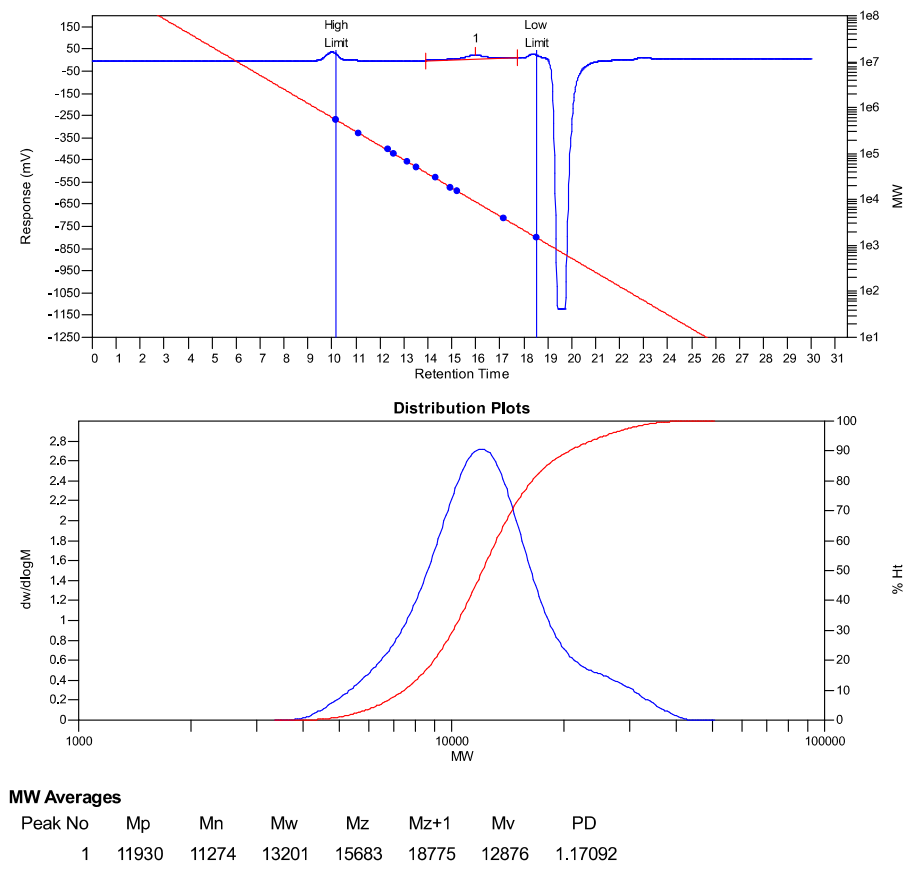


**Figure S8.** The GPC result for PhMA_33_-*b*-P(AEMA_17_-*g*-DOTA_8_).


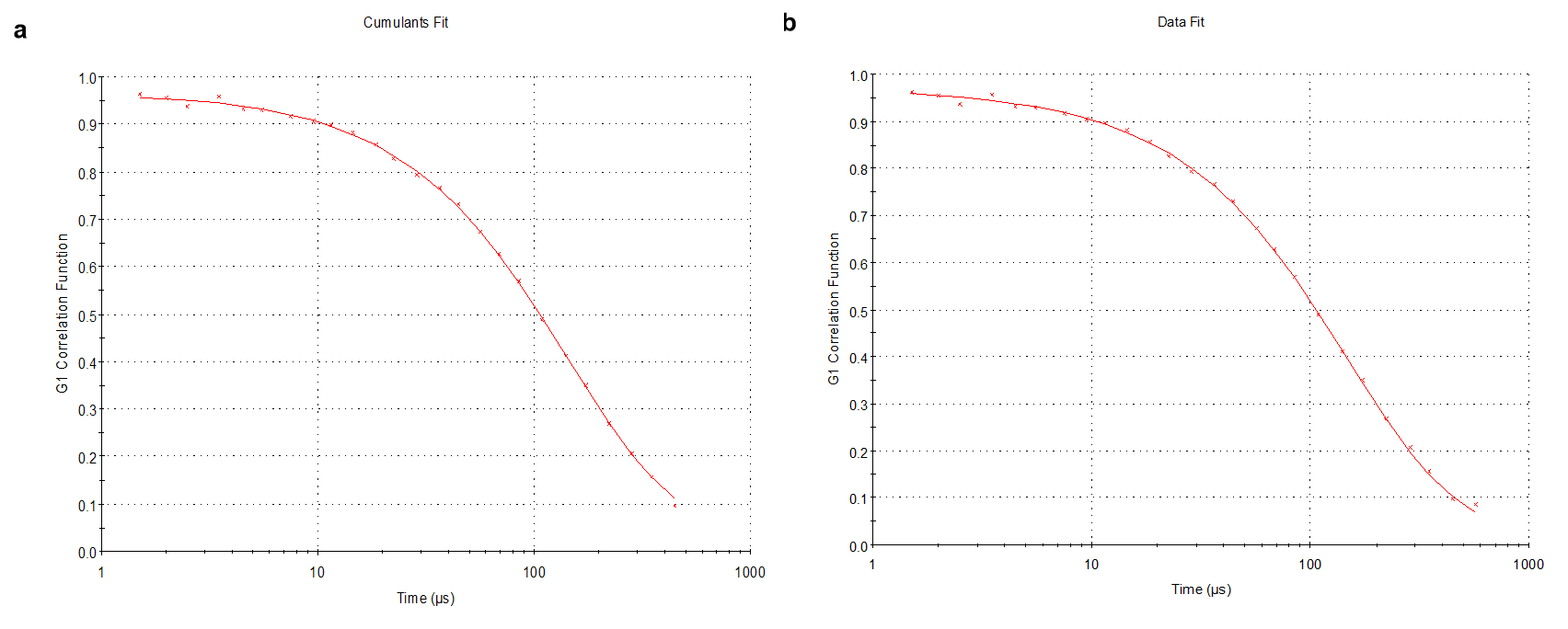


**Figure S9.** The mian autocorrelation plots of cumulants fit (M) (a) and distribution fit (M) (b) for calculating the hydrodynamic size of MRRA_N_ .

**Table S1**. The structure parameters of PAEMA_25_, PAEMA_16_-*g*-DOTA_9_, PPhMA_33_-*b*-PAEMA_25_, and PPhMA_33_-*b*-P(AEMA_17_-*g*-DOTA_8_).

| Samples | DP^a^ | *M*_n, NMR_ (KDa)^b^ | *M*_n,_ _GPC_ (KDa)^c^ | *M*_w_ / *M*_n_^c^ |
| --- | --- | --- | --- | --- |
| PAEMA_25_ | 0 | 4.4 | 3.9 | 1.09 |
| PAEMA_16_-*g*-DOTA_9_ | 9 | 8.9 | 5 | 1.11 |
| PPhMA_33_-*b*-PAEMA_25_ | 0 | 11.4 | 9.6 | 1.37 |
| PPhMA_33_-*b*-P(AEMA_17_-*g*-DOTA_8_) | 8 | 15.4 | 11.3 | 1.17 |

^a^ Averaged polymerization degree for grafted DOTA. ^b^ Number-average molecular weights determined by ^1^H NMR. ^c^ molecular weights and molecular weight distributions, *M*_w_ / *M*_n_, were evaluated by GPC.


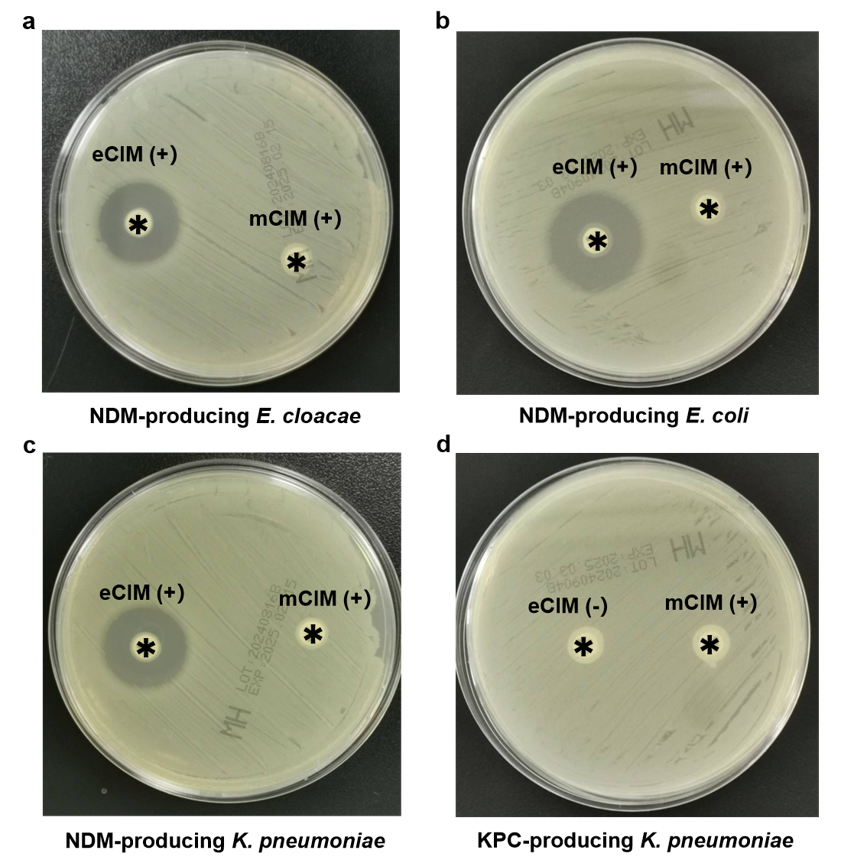


**Figure S10.** a-d) The screening for NDM-producing *E. cloacae*, NDM-producing *E. coli*, NDM-producing *K. pneumoniae* and KPC-producing *K. pneumoniae* based on mCIM and eCIM according to the CLSI M100-32. ✱ represents MEM (10 μg).

**
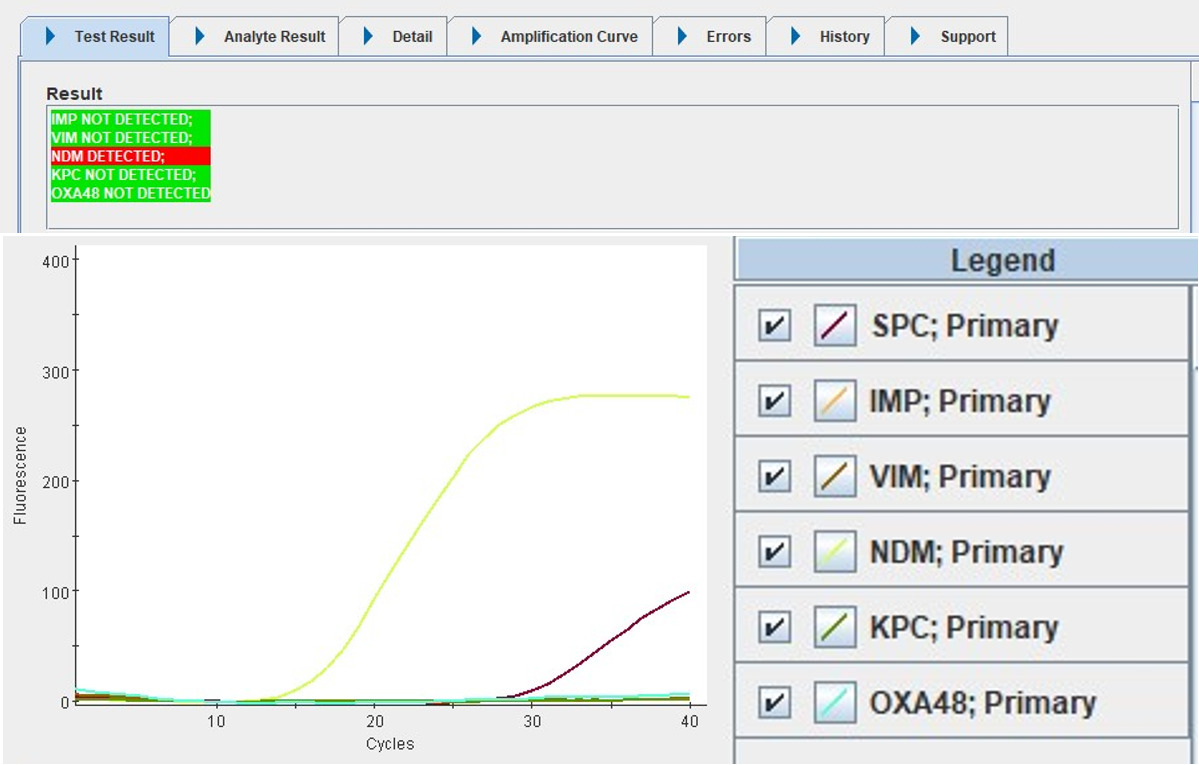
**

**Figure S11.** The confirmation of NDM resistance gene based on fluorescent PCR technology for NDM-producing *E. cloacae* conducted on GeneXpert® Infinity Systems.


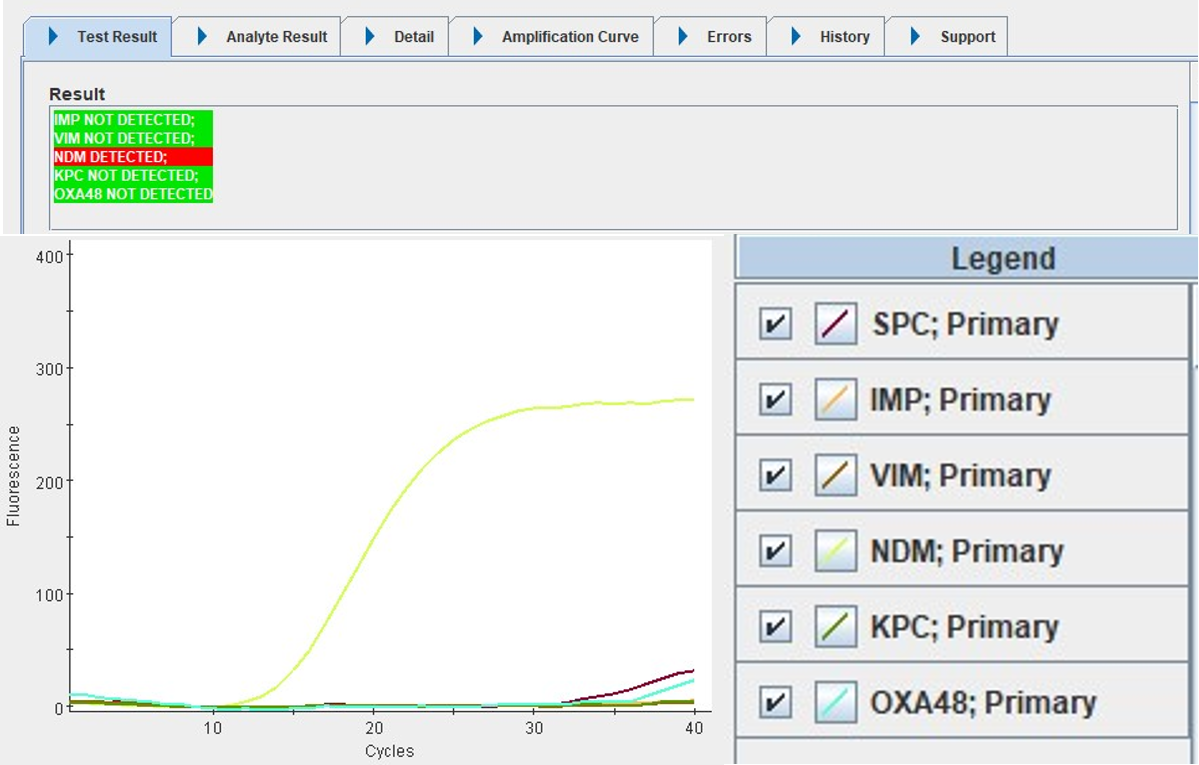


**Figure S12.** The confirmation for NDM resistance gene based on fluorescent PCR technology for NDM-producing *E. coli* conducted on GeneXpert® Infinity Systems.

**
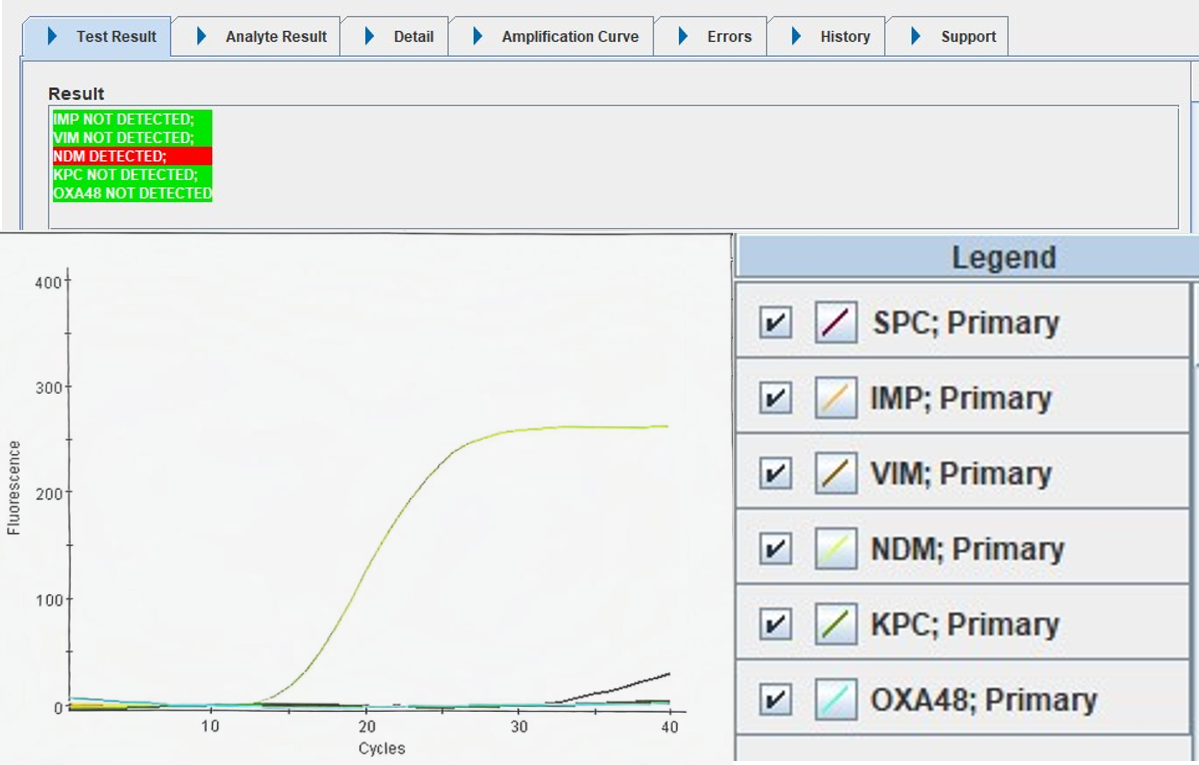
**

**Figure S13.** The confirmation for NDM resistance gene based on fluorescent PCR technology for NDM-producing *K. pneumoniae* conducted on GeneXpert® Infinity Systems.

**
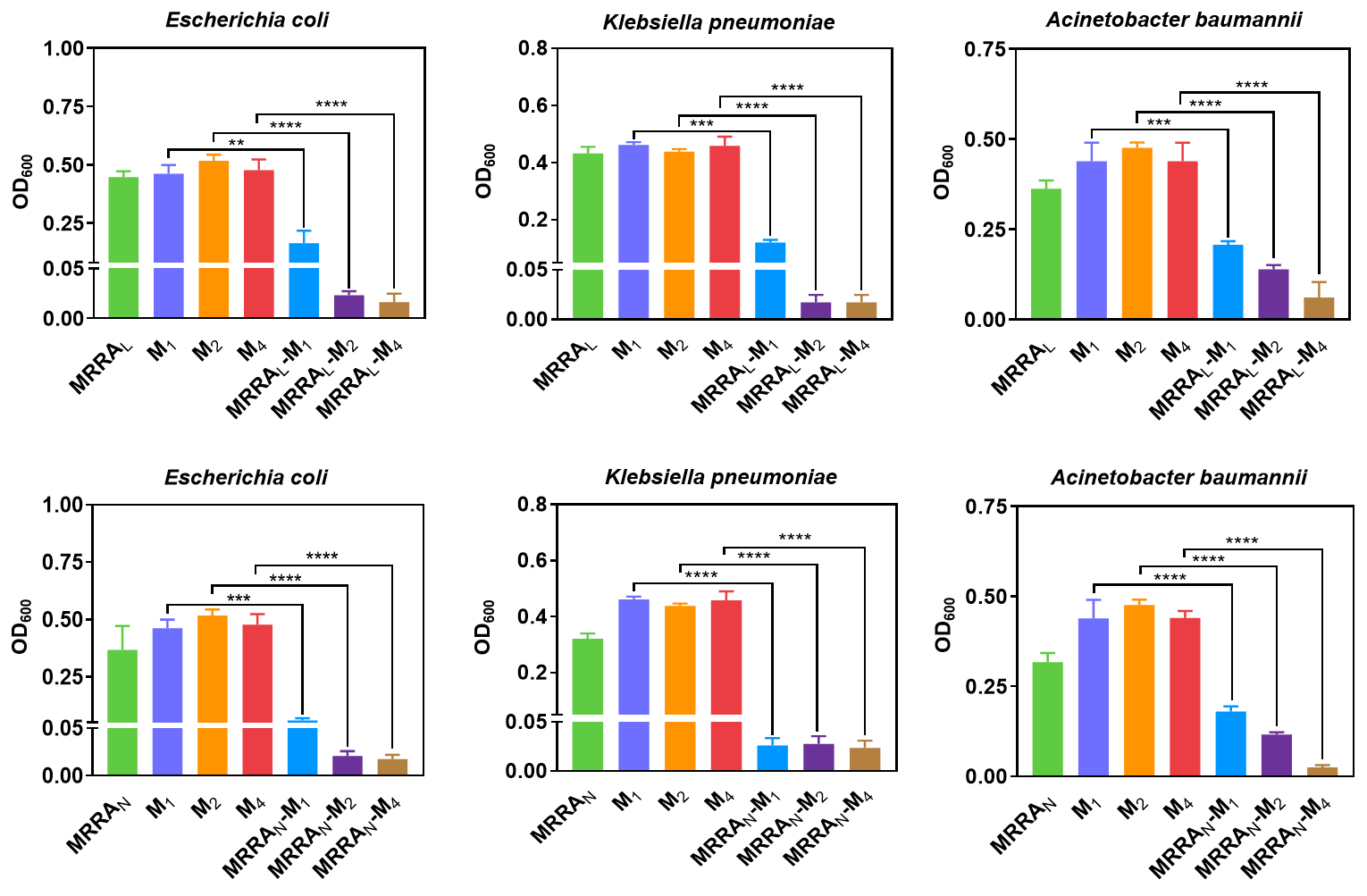
**

**Figure S14.** The OD_600_ of the bacteria treated with or without of MRRA_L_ (5.60 μM) / MRRA_N_ (self-assembled by 5.60 μM of PPhMA_33_-*b*-P(AEMA_17_-*g*-DOTA_8_) combined with different concentration of MEM (1 μg mL^−1^ (M_1_), 2 μg mL^−1^ (M_2_), and 4 μg mL^−1^ (M_4_)) against NDM-producing *E. coli*, NDM-producing *K. pneumoniae* and pan-drug-resistant *A. baumannii*, respectively. Data are presented as mean ± SD (n = 3); **, ***, and **** indicate *p* <0.01, *p* < 0.001, and *p* < 0.0001 respectively.


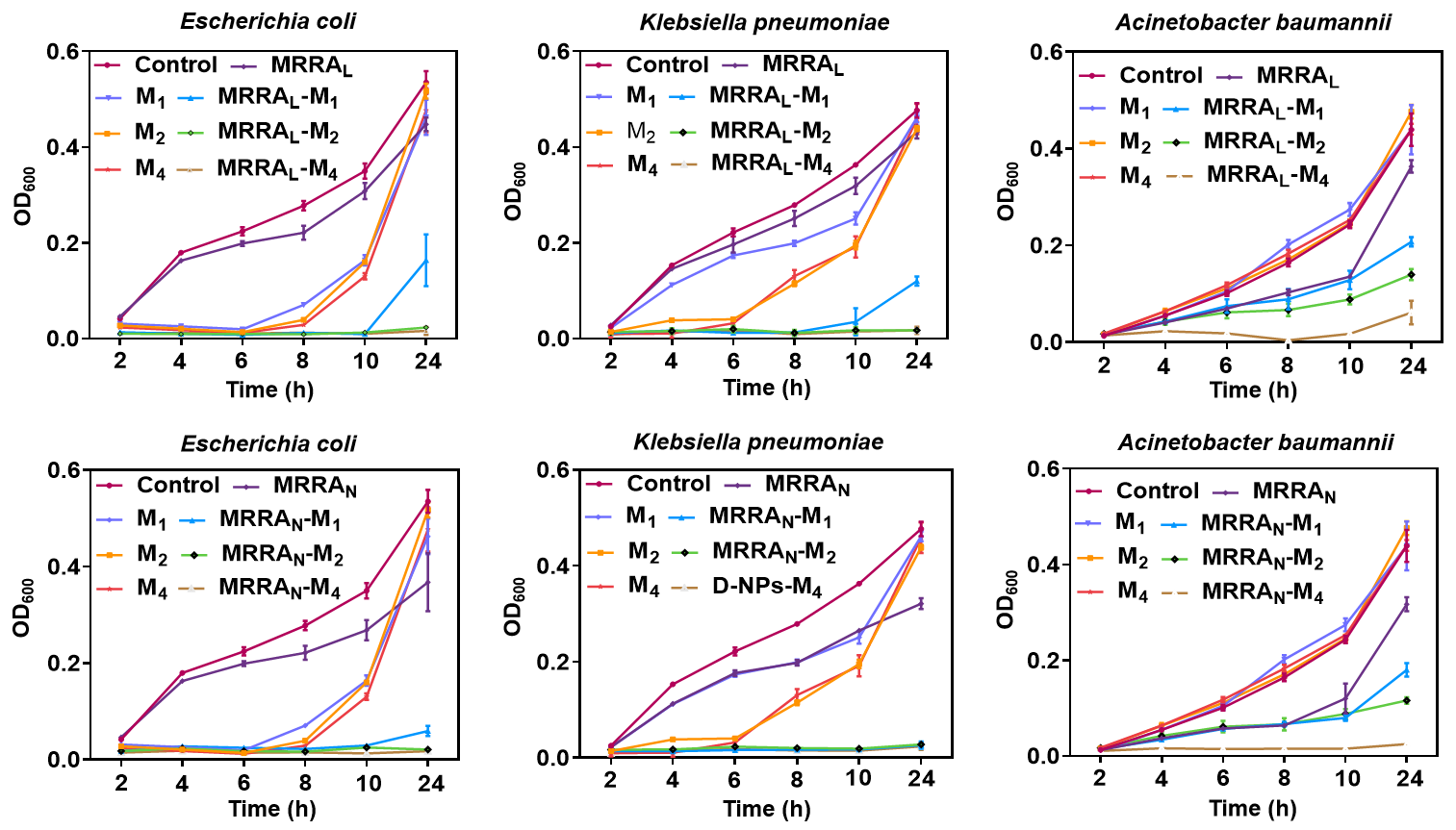


**Figure S15.** Time-dependent growth curves of bacteria treated with or without MRRA_L_ (5.60 μM) / MRRA_N_ (self-assembled by 5.60 μM of PPhMA_33_-*b*-P(AEMA_17_-*g*-DOTA_8_) combined with different concentration of MEM (1 μg mL^−1^ (M_1_), 2 μg mL^−1^ (M_2_), and 4 μg mL^−1^ (M_4_)) against NDM-producing *E. coli*, NDM-producing *K. pneumoniae* and pan-drug-resistant *A. baumannii*.


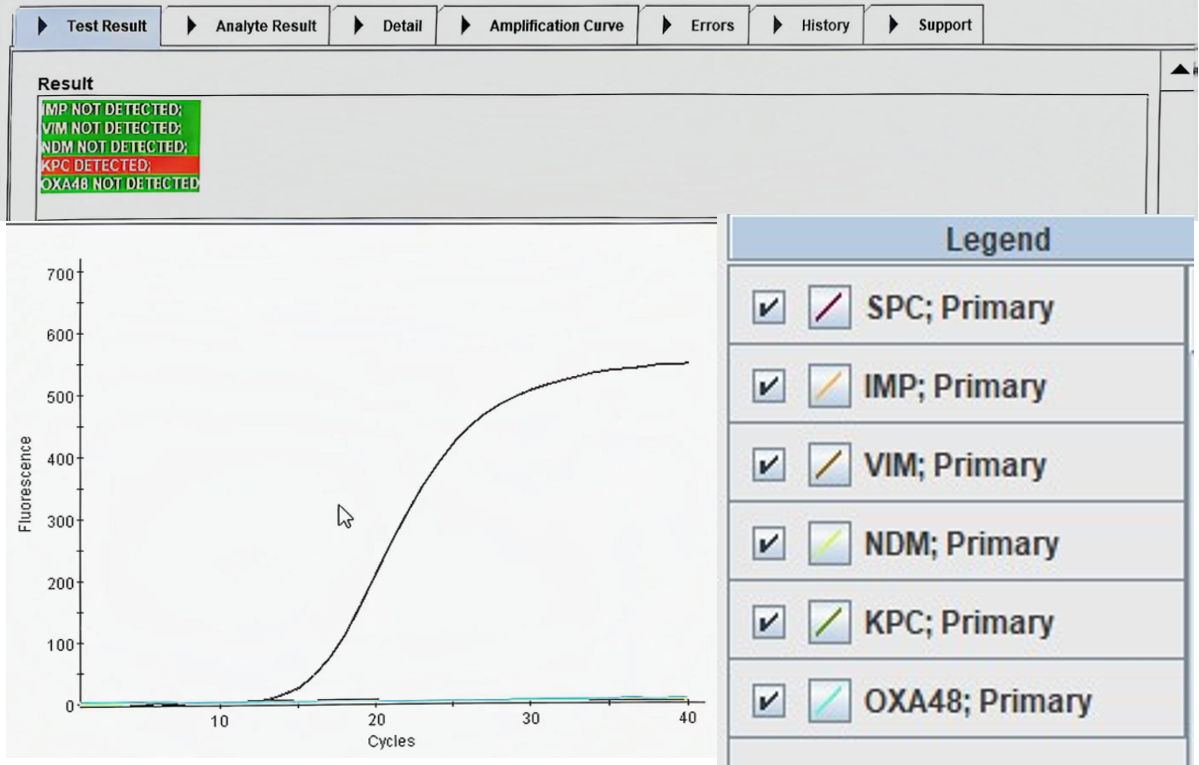


**Figure S16.** The confirmation for KPC resistance gene based on fluorescent PCR technology for KPC-producing *K. pneumoniae* conducted on GeneXpert® Infinity Systems.

**Table S2**. The MIC_MEM_ combined with MRRA_L_/MRRA_N_ (5.60 μM) for every evolved subpopulation of NDM-producing *E. cloacae*.

| **Passage No.** | CMIC_MEM_ (μg mL^-1^) ^a^ | |
| --- | --- | --- |
|  | MRRA_L_-M | MRRA_N_-M |
| 1 | 1 | 0.5 |
| 2 | 1 | 0.5 |
| 3 | 1 | 0.5 |
| 4 | 1 | 0.5 |
| 5 | 1 | 0.5 |
| 6 | 1 | 0.5 |
| 7 | 2 | 0.5 |
| 8 | 2 | 0.5 |
| 9 | 2 | 1 |
| 10 | 2 | 1 |
| 11 | 2 | 1 |
| 12 | 2 | 1 |

^a^ The MIC_MEM_ combined with MRRA_L_/MRRA_N_.


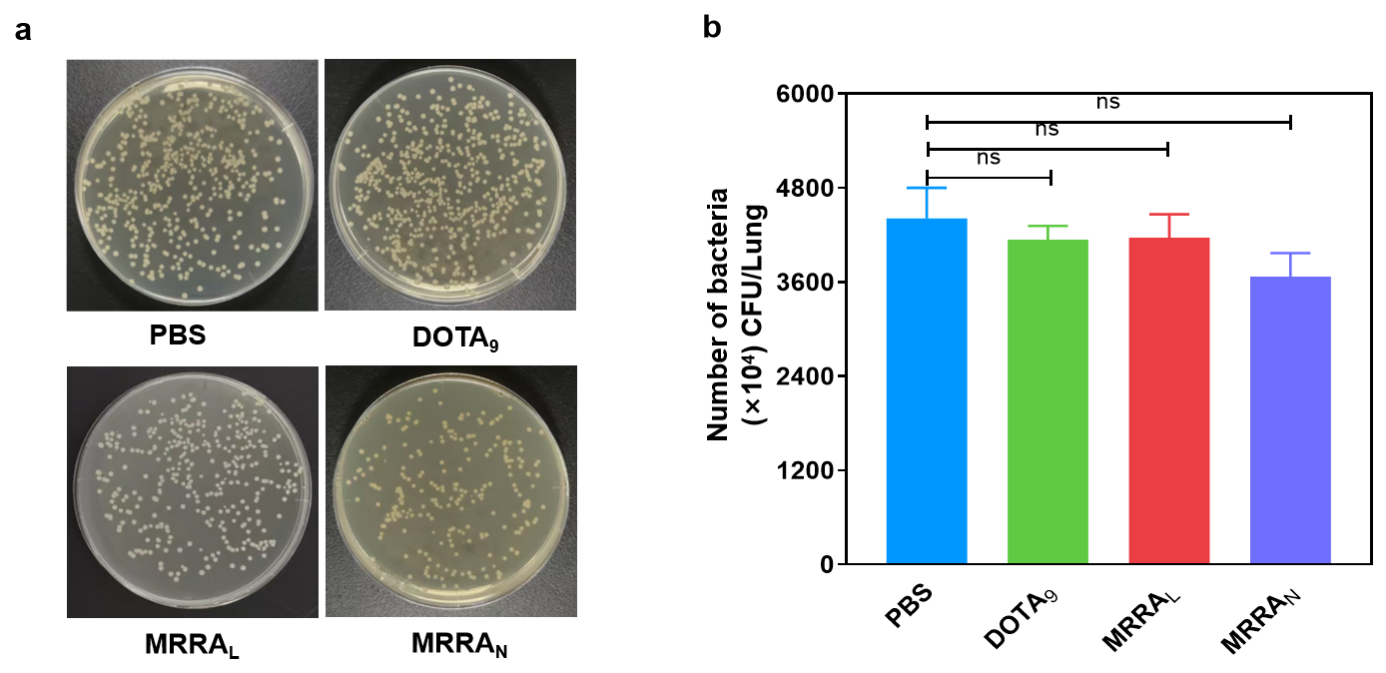


**Figure S17.** a) Images of typical agar plates of bacterial loads of lung homogenates treated with monotherapy of PBS, DOTA_9_, MRRA_L_ and MRRA_N_. b) The corresponding number of bacteria in lung homogenates receiving the same therapeutic process. Data are presented as mean ± SD (n = 5); ns stands for nonsignificant.


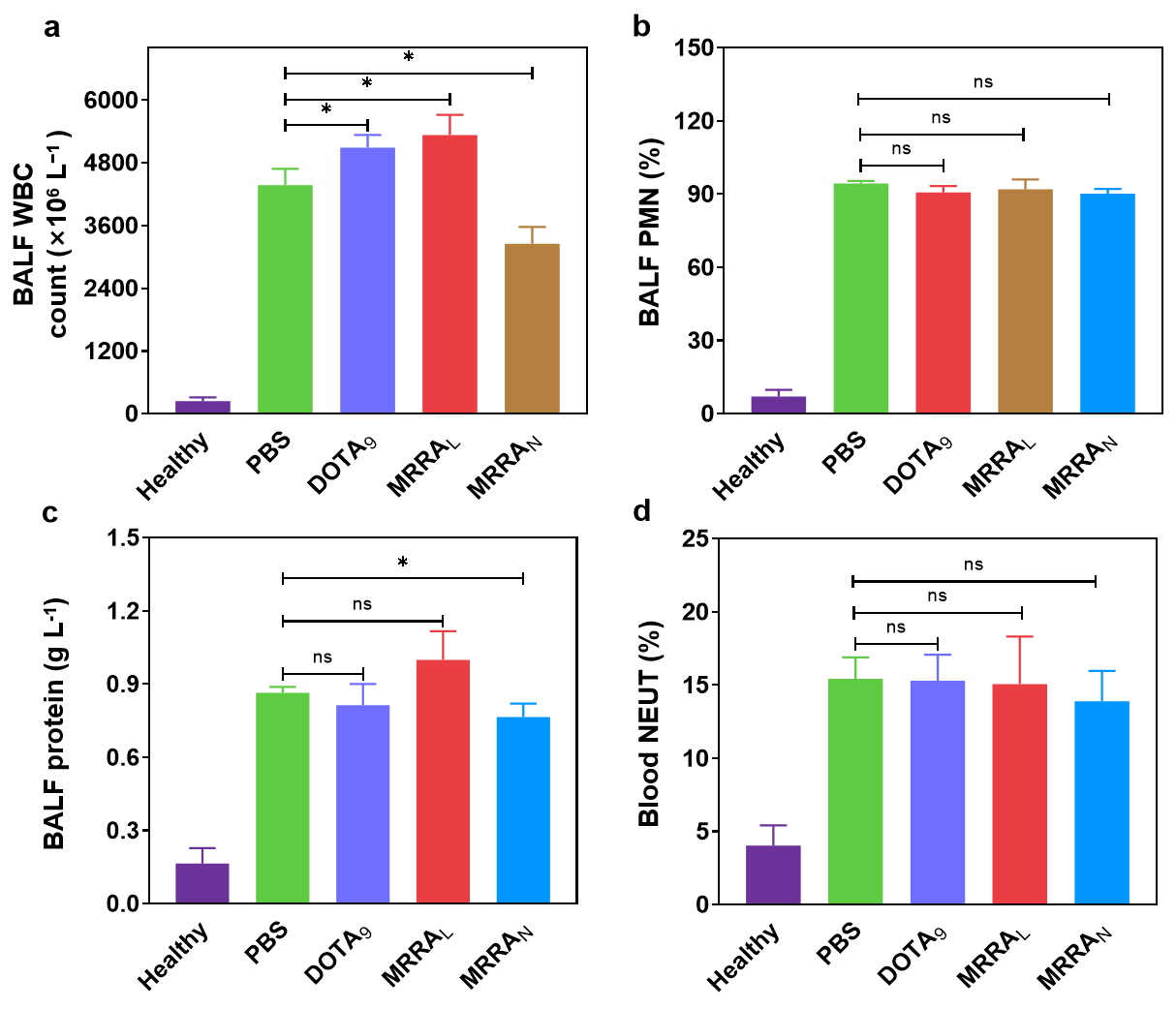


**Figure S18.** a-c) The total WBC, PMN (%), and total protein content in BALF treated with monotherapy of PBS, DOTA_9_, MRRA_L_ and MRRA_N_, respectively. d) The NEUT (%) in blood receiving the same therapeutic process. Data are presented as mean ± SD (n = 5); ns stands for nonsignificant, *indicate *p* <0.05.


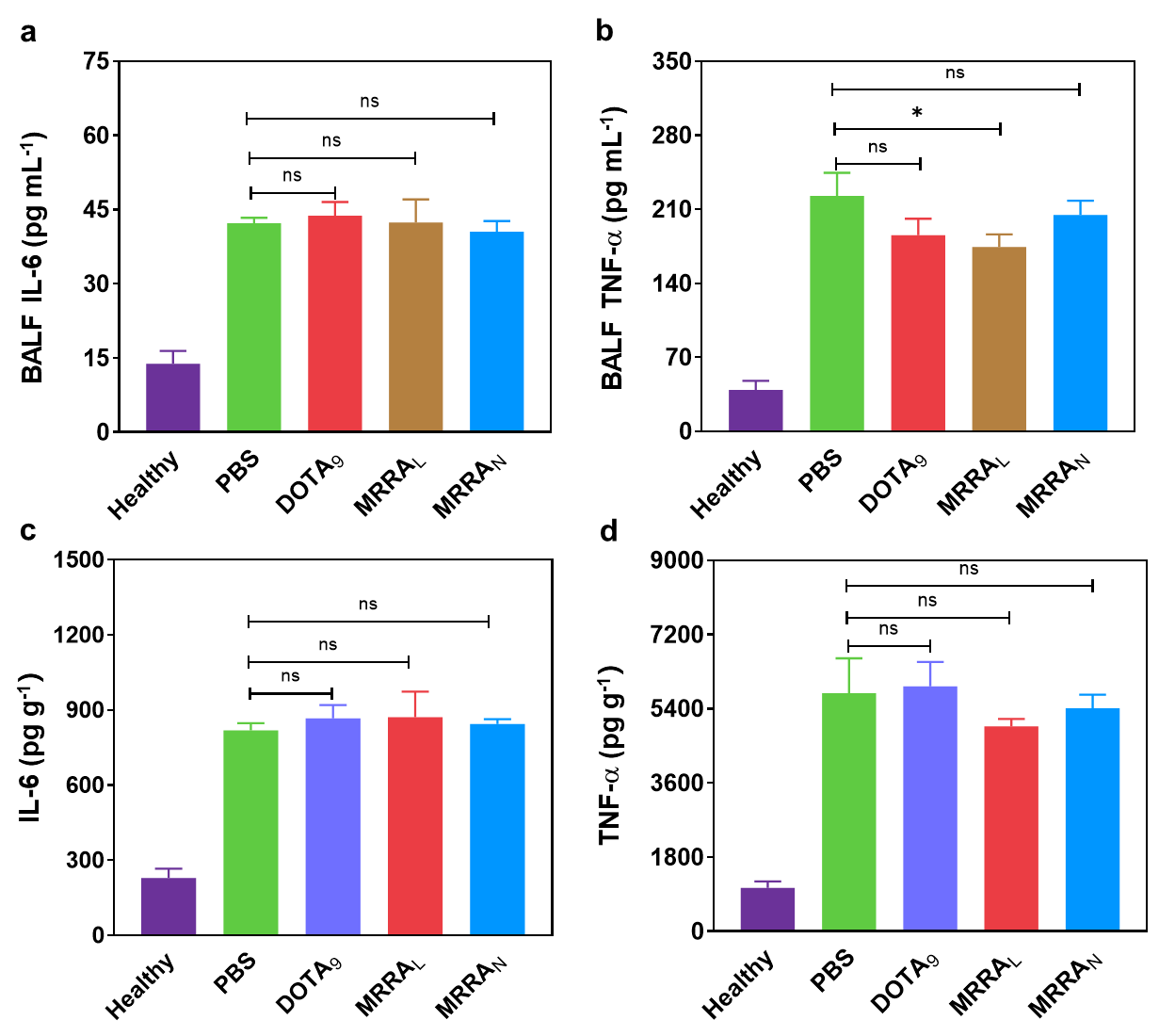


**Figure S19.** a-b) The levels of proinflammatory cytokines (IL-6, TNF-α) in BALF treated with monotherapy of PBS, DOTA_9_, MRRA_L_ and MRRA_N_, respectively. c-d) The levels of proinflammatory cytokines (IL-6, TNF-α) in lung tissue homogenate supernatant receiving the same therapeutic process. Data are presented as mean ± SD (n = 5); ns stands for nonsignificant, *indicate *p* <0.05.


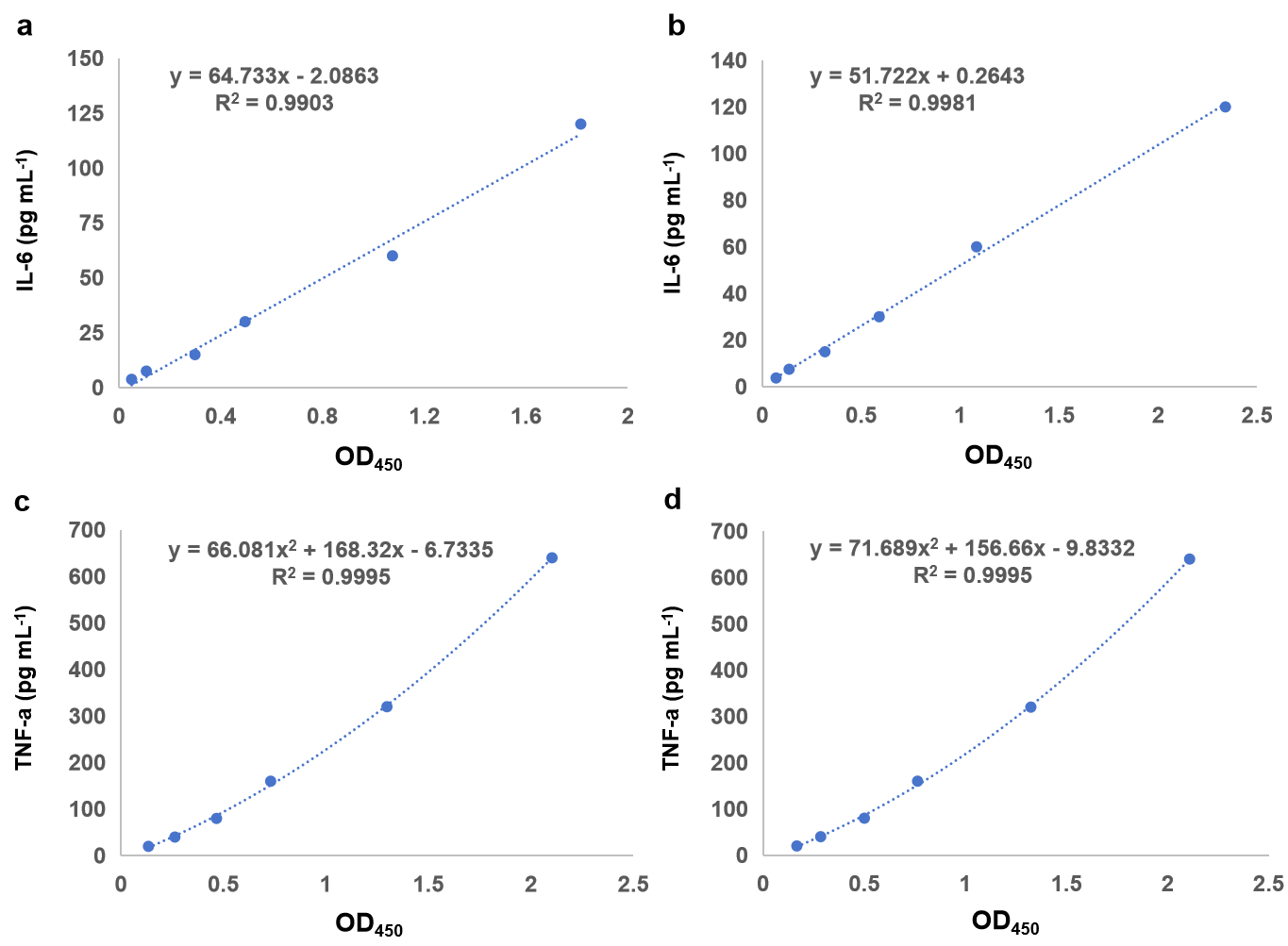


**Figure S20.** The standard curves of IL-6 for BALF (a) and lung tissue homogenate supernatant (b). The standard curves of TNF-α for BALF (c) and lung tissue homogenate supernatant (d).


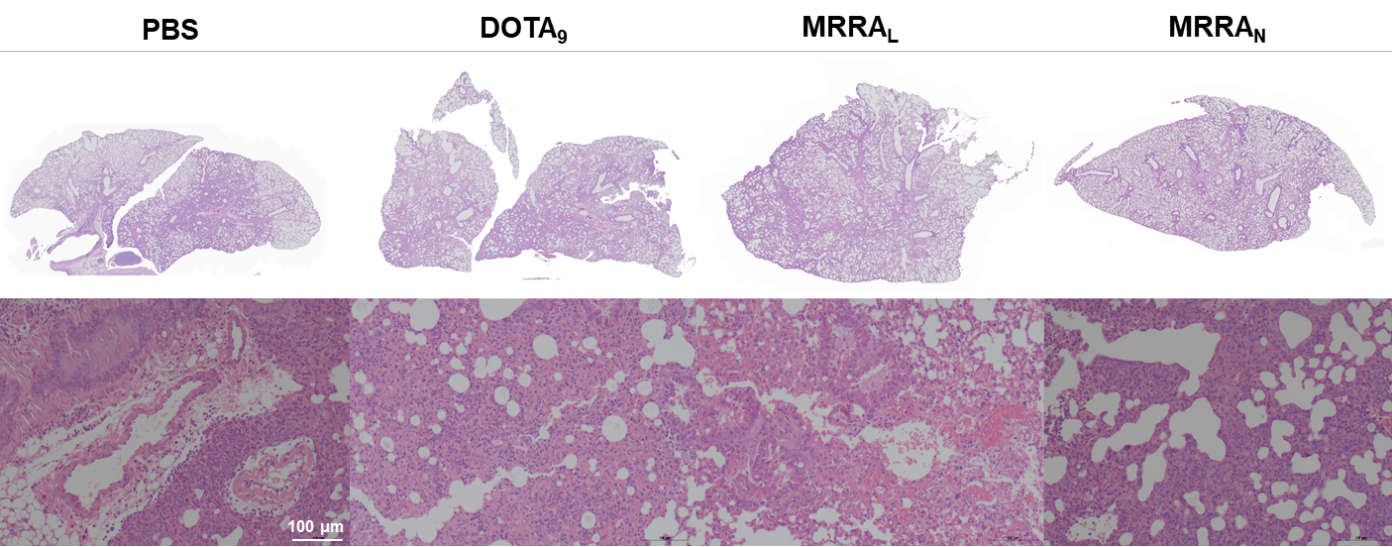


**Figure S21.** Hematoxylin and eosin (H&E) staining of lung tissues for groups of PBS, DOTA_9_, MRRA_L_, and MRRA_N_ monotherapy, respectively. Scale bar = 100 μm.


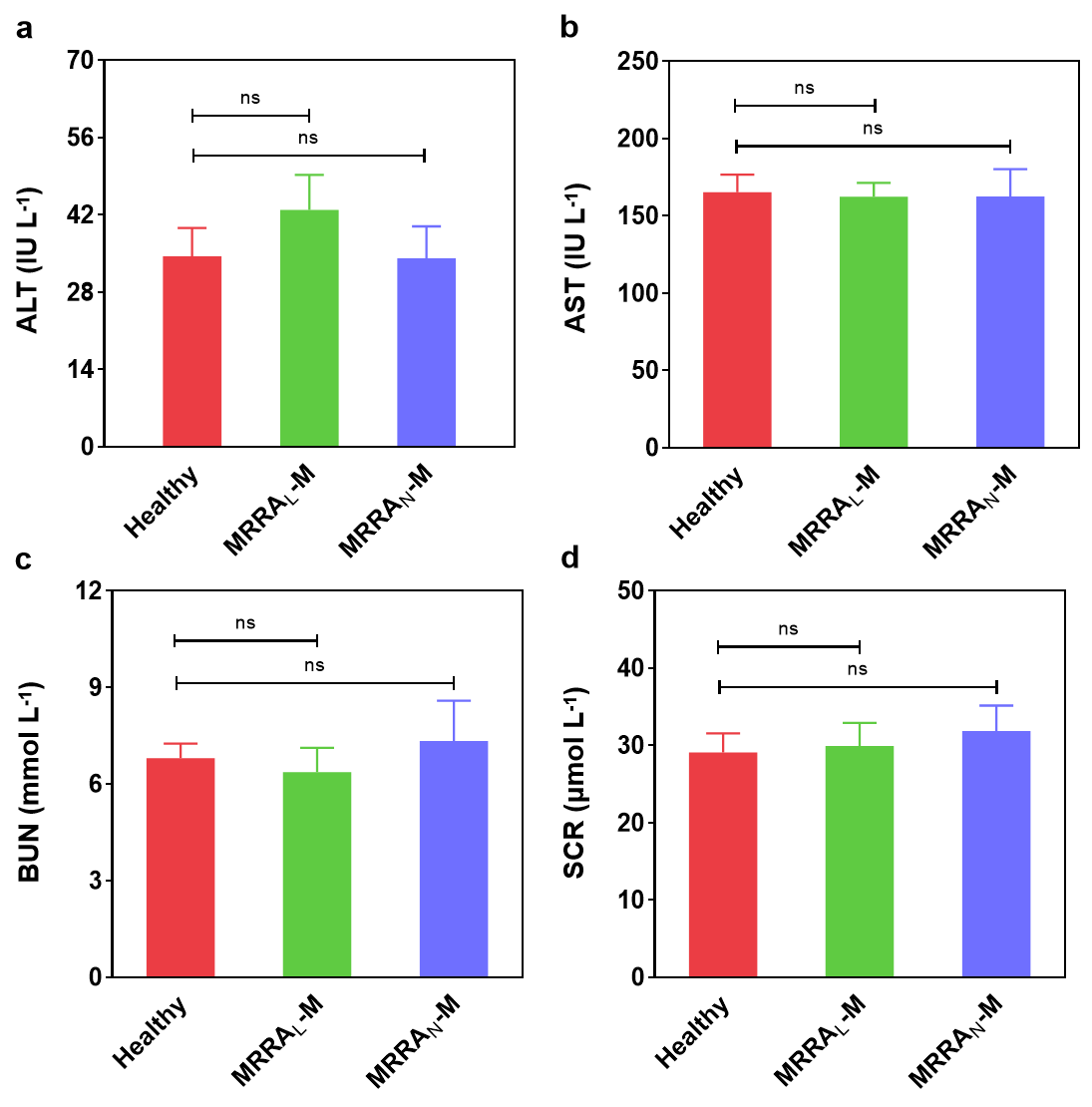


**Figure S22.** Biosafety evaluation for main hepatic function and renal function of ALT, AST, BUN and SCR, respectively. Data are presented as mean ± SD (n = 5); ns stands for nonsignificant.


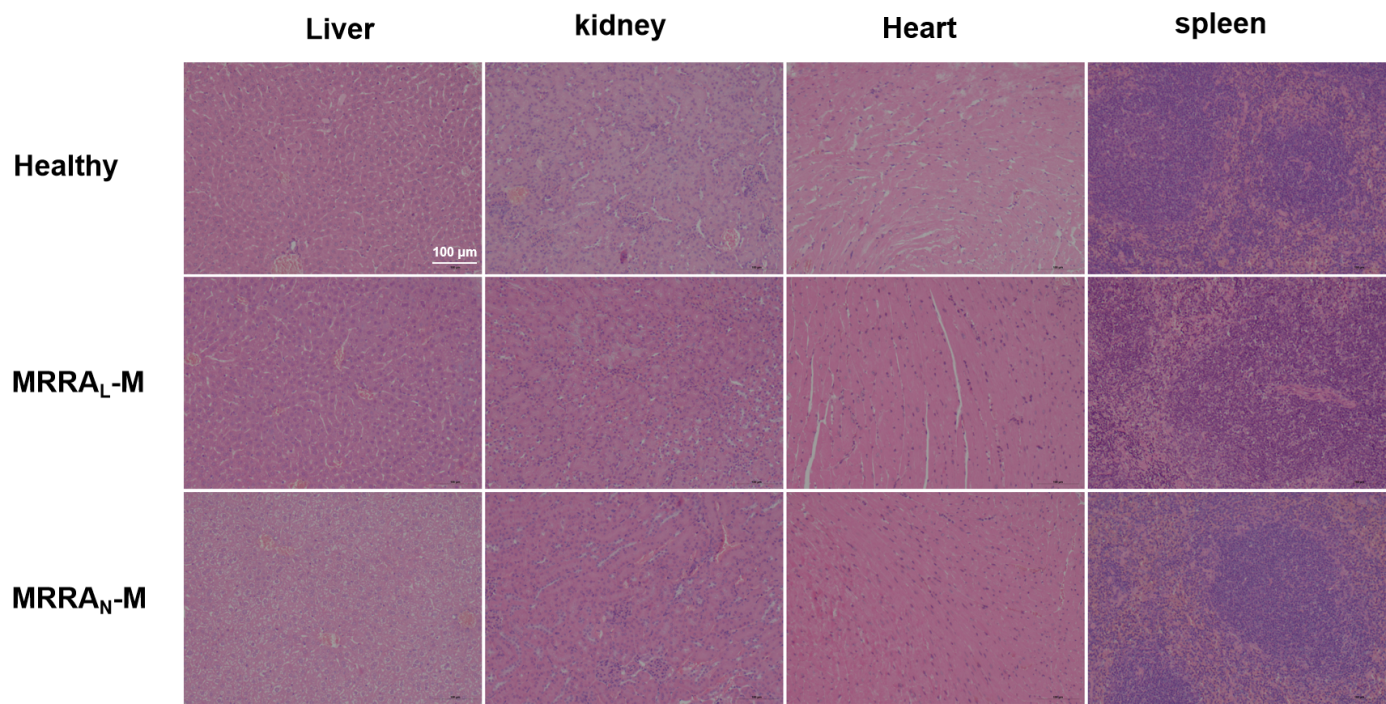


**Figure S23.** Biosafety evaluation of tissues section for liver, kidney, heart and spleen in co-therapy groups of MRRA_L_-M and MRRA_N_-M using healthy group as control. Scale bar = 100 μm.
